# Inorganic Salts of *N*-phenylbiguanidium(1+)—Novel Family with Promising Representatives for Nonlinear Optics

**DOI:** 10.3390/ijms22168419

**Published:** 2021-08-05

**Authors:** Irena Matulková, Ivana Císařová, Michaela Fridrichová, Róbert Gyepes, Petr Němec, Jan Kroupa, Ivan Němec

**Affiliations:** 1 Department of Inorganic Chemistry, Faculty of Science, Charles University, Hlavova 8, 128 43 Prague, Czech Republic; irena.matulkova@natur.cuni.cz (I.M.); ivana.cisarova@natur.cuni.cz (I.C.); michaela.fridrichova@natur.cuni.cz (M.F.); 2 Department of Molecular Electrochemistry and Catalysis, J. Heyrovský Institute of Physical Chemistry, Czech Academy of Sciences, Dolejškova 3, 182 23 Prague, Czech Republic; robert.gyepes@jh-inst.cas.cz; 3 Department of Chemical Physics and Optics, Faculty of Mathematics and Physics, Charles University, Ke Karlovu 3, 121 16 Prague, Czech Republic; nemec@karlov.mff.cuni.cz; 4 Department of Dielectrics, Institute of Physics, Czech Academy of Sciences, Na Slovance 2, 182 21 Prague, Czech Republic; kroupa@fzu.cz

**Keywords:** *N*-phenylbiguanidium(1+) cation, crystal structure, vibrational spectra, second harmonic generation, solid-state DFT

## Abstract

Seven inorganic salts containing *N*-phenylbiguanide as a prospective organic molecular carrier of nonlinear optical properties were prepared and studied within our research of novel hydrogen-bonded materials for nonlinear optics (NLO). All seven salts, namely *N*-phenylbiguanidium(1+) nitrate (*C*2/*c*), *N*-phenylbiguanidium(1+) perchlorate (*P*-1), *N*-phenylbiguanidium(1+) hydrogen carbonate (*P*2_1_/*c*), bis(*N*-phenylbiguanidium(1+)) sulfate (*C*2), bis(*N*-phenylbiguanidium(1+)) hydrogen phosphate sesquihydrate (*P*-1), bis(*N*-phenylbiguanidium(1+)) phosphite (*P*2_1_), and bis(*N*-phenylbiguanidium(1+)) phosphite dihydrate (*P*2_1_/*n*), were characterised by X-ray diffraction (powder and single-crystal X-ray diffraction) and by vibrational spectroscopy (FTIR and Raman). Two salts with non-centrosymmetric crystal structures—bis(*N*-phenylbiguanidium(1+)) sulfate and bis(*N*-phenylbiguanidium(1+)) phosphite—were further studied to examine their linear and nonlinear optical properties using experimental and computational methods. As a highly SHG-efficient and phase-matchable material transparent down to 320 nm and thermally stable to 483 K, bis(*N*-phenylbiguanidium(1+)) sulfate is a promising novel candidate for NLO.

## 1. Introduction

In addition to inorganic materials for nonlinear optics (NLO) (e.g., binary and ternary compounds, salts, transition-metals complexes, and tungsten bronze oxide materials, among others [1,2,3,4,5]), polarisable organic molecules and their salts and cocrystals stand out as an alternative and highly promising class of NLO materials [6,7,8,9]. NLO materials are widely used as efficient optical elements for frequency shifting, signal processing, and optical communication, among other functions. Moreover, several molecular crystals exhibit various *χ*^(2)^ and *χ*^(3)^ NLO effects (including second harmonic generation (SHG), third harmonic generation (THG), and cascaded self-frequency doubling and tripling) in addition to being promising stimulated Raman scattering (SRS)-active materials [10,11].

The most efficient way of designing and preparing these molecular materials consists of using a two-step approach, as described previously in [12]: (a) molecular engineering, i.e., synthesis (selection) of a proper molecular carrier with NLO properties and (b) crystal structure engineering, i.e., using suitable intermolecular interactions and/or co-crystallisation partners to grow crystals with the desired symmetry and NLO properties. The symmetry of these molecules and proper crystal class is crucial, especially for *χ*^(2)^ NLO effects (e.g., SHG), where the absence of an inversion centre is the symmetry requirement.

Crystal engineering of structures containing polar organic molecules mostly utilises hydrogen bonding as the dominant inter-molecular interaction for adequate crystal packing, and the role of this bonding has been widely discussed in previous studies [12,13,14,15,16,17]. Furthermore, implementing a suitable hydrogen-bonded counter molecule/ion can counteract undesirable tendencies of the selected organic molecules (cations) to form centrosymmetric pairs. The concept [18], based on the use of chains/networks formed by inorganic hydrogen-bonded anions (i.e., anions acting as hydrogen bonds donors), was successfully applied to several salts and cocrystals prepared in our laboratory, e.g., references [19,20,21,22]. However, the presence of deprotonated anions acting as strict hydrogen bond acceptors can also lead to the formation of promising non-centrosymmetric phases, e.g., references [19,23,24,25].

Biguanides and their derivatives are an interesting class of compounds not only for their extensive medicinal applications but also for their ability to form stable metal complexes and intermediates useful for heterocycles synthesis and organocatalysis. Their applications and structural characteristics have been recently reviewed [26]. Furthermore, biguanides and their derivatives, such as *N*-phenylbiguanides, have been considered promising building blocks in crystal engineering. Crystals containing their cations have been studied as potential proton transfer materials [27,28] and prospective materials for NLO [24,29,30]. DFT computations on the molecular level indicate that biguanidium and *N*-phenylbiguanidium cations exhibit promising values of hyperpolarisability [29,30,31], as confirmed by the experimental determination of SHG efficiency, especially for inorganic salts of biguanide [24].

The aim of this paper is to extend the small family of *N*-phenylbiguanidium(1+) crystalline salts [27,28,32,33,34] by preparing novel members containing inorganic anions (as co-crystallisation partners) with diverse symmetries and hydrogen bonding potential for activating different self-assembly modes in crystal packing, thereby obtaining new, prospective molecular materials for NLO. For this purpose, seven novel compounds, *N*-phenylbiguanidium(1+) nitrate (**phbiguaNO_3_**), *N*-phenylbiguanidium(1+) perchlorate (**phbiguaClO_4_**), *N*-phenylbiguanidium(1+) hydrogen carbonate (**phbiguaHCO_3_**), bis(*N*-phenylbiguanidium(1+)) sulfate (**phbigua_2_SO_4_**), bis(*N*-phenylbiguanidium(1+)) hydrogen phosphate sesquihydrate (**phbigua_2_HPO_4_1.5H_2_O**), bis(*N*-phenylbiguanidium(1+)) phosphite (**phbigua_2_HPO_3_**), and bis(*N*-phenylbiguanidium(1+)) phosphite dihydrate (**phbigua_2_HPO_3_2H_2_O**) were prepared and characterised both structurally and spectroscopically. Lastly, the linear and nonlinear optical properties of two of these salts with desired non-centrosymmetric packing, **phbigua_2_SO_4_** and **phbigua_2_HPO_3_**, were studied and discussed.

## 2. Results and Discussion

### 2.1. Crystal Structures

The crystal structures were solved using the single-crystal X-ray diffraction method—basic crystallographic data of all studied salts are summarised in Table 1 and Table 2. Measurement and refinement details are presented in Tables S1 and S2, Supplementary Materials. Selected bond lengths and angles, including those of hydrogen bonds, are presented in Tables S3–S9, Supplementary Materials. All figures were created using the PLATON software [35].

**Figure 1:**
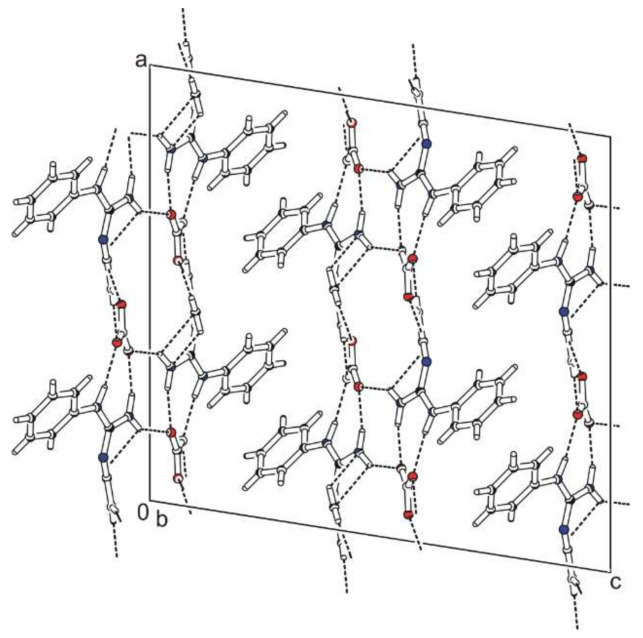
Packing scheme of **phbiguaNO_3_** (view along the *b* axis direction); dashed lines indicate hydrogen bonds, solid lines indicate unit cell.

*N*-phenylbiguanidium(1+) nitrate, **phbiguaNO_3_**, crystallises in the monoclinic system (*C*2/*c* space group). Atom numbering is shown in Figure S1, Supplementary Materials. The crystal structure is based on chains containing alternating cations and anions interconnected via N-H...O hydrogen bonds, which form double layers, as shown in Figure 1.

The chains in these layers can be described [36,37] by the graph set descriptor C(7). Phenyl rings of the cations are oriented outwards from these layers. All nitrate anions are hydrogen-bonded to four *N*-phenylbiguanidium(1+) cations. Two of these cations are involved in eight-membered hydrogen-bonded rings—graph set descriptor $R_{2}^{2}$(8) (hydrogen bonds N5-H5B…O1^c^, N4-H4A…O2^c^ and N1-H1…O2^a^, N2-H2B…O3^a^ with the N...O distance ranging from 2.893(2) to 3.031(2) Å). The remaining cations are involved in one interaction, i.e., N4-H4B…O1^d^ or N2-H2A…O3^b^ with N…O distances 2.926(2) Å and 2.947(2) Å, respectively.

**Figure 2:**
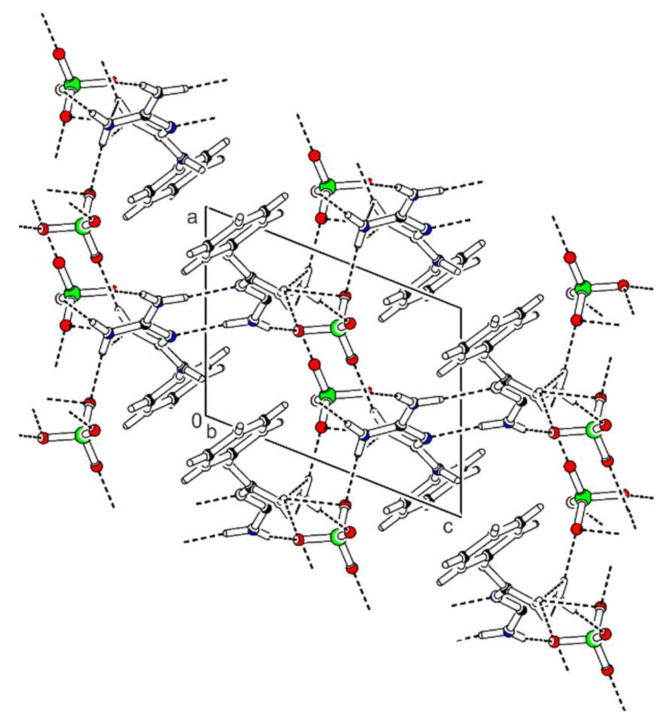
Packing scheme of **phbiguaClO_4_** (view along the *b* axis direction); dashed lines indicate hydrogen bonds, solid lines indicate unit cell.

*N*-phenylbiguanidium(1+) perchlorate, **phbiguaClO_4_**, crystallises in the triclinic system (space group *P*-1). Atom numbering is shown in Figure S2, Supplementary Materials. The crystal structure consists of centrosymmetric pairs of cations involved in N-H...O interactions with perchlorate anions, arranged in layers parallel to the *ab* plane, as shown in Figure 2. Each anion interacts with four neighbouring cations. Two of them are involved in simple N-H…O interactions (N4-H4A...O3^b^ and N2-H2A...O1 with N…O distances of 3.046(6) and 2.850(6) Å, respectively). The third cation is interconnected by bifurcated hydrogen bonds O3^a^...H2B(-N2)...O4^a^—graph set descriptor ${\;R}_{1}^{2}$(4)—and the fourth cation by the pair of hydrogen bonds N4-H4B...O2^c^ and N5-H5A...O4^c^ (N…O distance 2.962(6) and 3.025(6) Å, respectively)—graph set descriptor $R_{2}^{2}$(8). The *N*-phenylbiguanidium(1+) cations form pairs via intermolecular N-H…N interactions with an N…N distance of 3.006(4) Å and graph set descriptor $R_{2}^{2}$(8).

**Figure 3:**
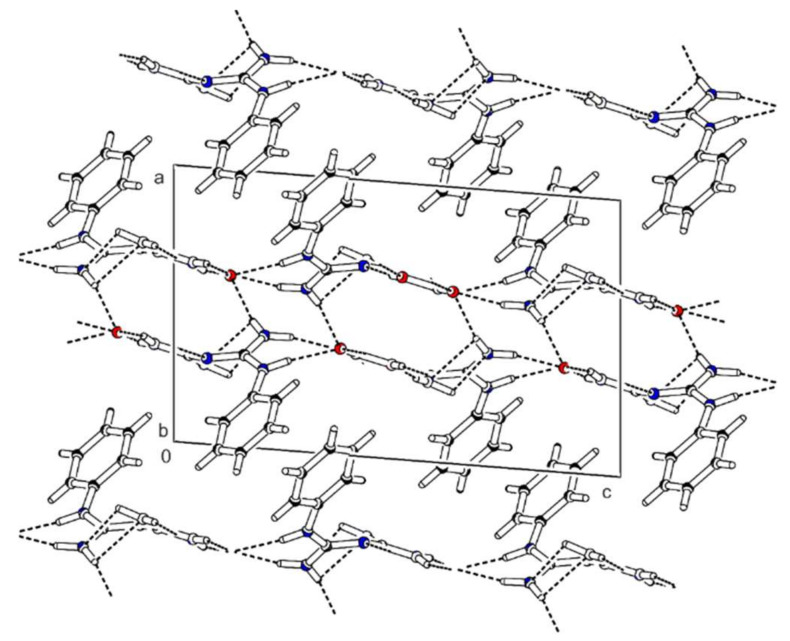
Packing scheme of **phbiguaHCO_3_** (view along the *b* axis direction); dashed lines indicate hydrogen bonds, solid lines indicate unit cell.

*N*-phenylbiguanidium(1+) hydrogen carbonate, **phbiguaHCO_3_**, crystallises in the monoclinic system (space group *P*2_1_/*c*). The atom numbering is shown in Figure S3, Supplementary Materials. The crystal structure contains double layers parallel to the *bc* plane formed by hydrogen bonded chains of alternating cations and anions (graph set descriptor C(8)) (see Figure 3). These chains are interconnected through N-H…O hydrogen bonds (N2-H2A…O2^b^ with an N...O distance of 2.873(1) Å) within the double layers. The resulting large hydrogen-bonded rings can be described using the graph set descriptor $R_{4}^{2}$(16). Phenyl rings of the cations are oriented outwards from these double layers. Each anion interacts with four neighbouring cations. For three cations, these interactions are provided by the pairs of N-H…O hydrogen bonds (N...O distance ranging from 2.806(1) to 2.878(1) Å) or by the combination of N-H…O and O-H…N hydrogen bonds (N5-H5A…O2^d^ and O1-H10…N3^a^ with N…O distances of 2.832(1) and 2.794(1) Å, respectively). The resulting hydrogen-bonded rings correspond to the graph set descriptor $R_{2}^{2}$(8). The last cation is involved in the aforementioned inter-chain interaction N2-H2A…O2^b^.

**Figure 4:**
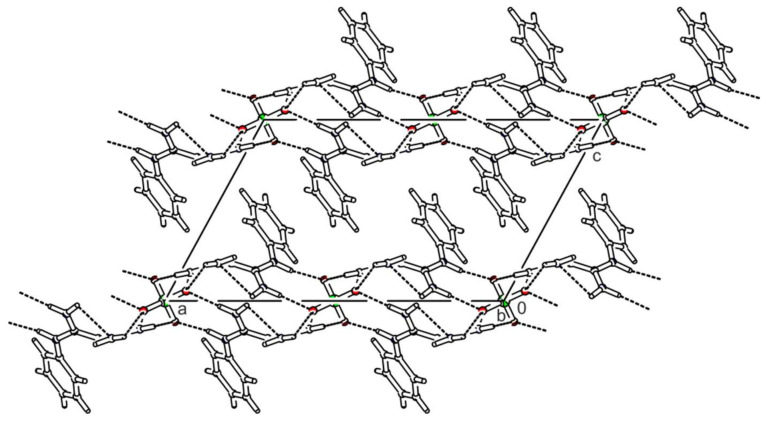
Packing scheme of **phbigua_2_SO_4_** (view along the *b* axis direction); dashed lines indicate hydrogen bonds, solid lines indicate unit cell.

Bis(*N*-phenylbiguanidium(1+)) sulfate, **phbigua_2_SO_4_**, crystallises in the monoclinic system (non-centrosymmetric space group *C*2). Atom numbering is shown in Figure S4, Supplementary Materials. The crystal structure is based on layers (parallel to the *ab* plane) which consist of alternating cations and anions (see Figure 4) interconnected by five types of N-H...O hydrogen bonds. In these layers, the arrangement can be described by graph set descriptor C(3). The phenyl rings of the *N*-phenylbiguanidium(1+) cations are oriented outwards from the layers. Each anion interacts with five neighbouring cations. Four of them are involved in eight-membered hydrogen-bonded rings formed by the pairs of N-H...O hydrogen bonds (N1-H1…O2 and N2-H2B…O1, N4-H4A…O1^a^ and N5-H5B…O2^c^, with N…O distances ranging from 2.813(3) to 3.066(4) Å, graph set descriptors $R_{2}^{2}$(8)). The remaining two cations are connected via a single N5-H5A...O1^b^ hydrogen bond (N…O distances 2.849(4) Å).

**Figure 5:**
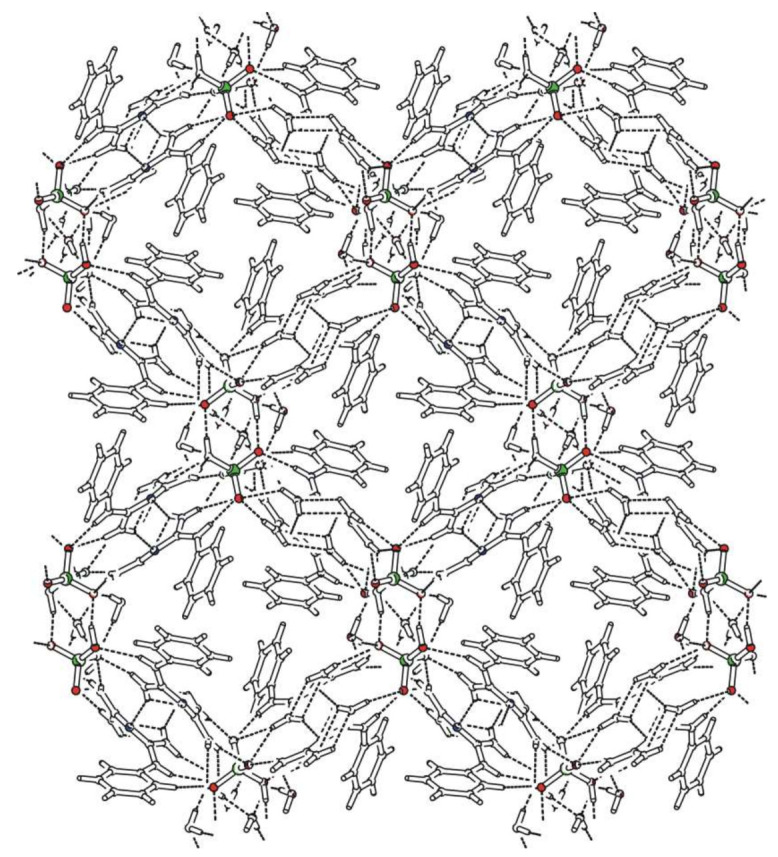
Packing scheme of **phbigua_2_HPO_4_1.5H_2_O** (view along the *a* axis direction); dashed lines indicate hydrogen bonds.

Bis(*N*-phenylbiguanidium(1+)) hydrogen phosphate sesquihydrate, **phbigua_2_HPO_4_1.5H_2_O**, crystallises in the triclinic system (space group *P*-1). The asymmetric unit contains eight *N*-phenylbiguanidium(1+) cations, six molecules of water, and four hydrogen phosphate anions. The cations form chains (graph set descriptor C(4)) oriented along the *a*-axis via intermolecular N-H...N hydrogen bonds (with N...N distances ranging from 3.033(2) to 3.220(3) Å). The anions form pairs interconnected by O-H…O hydrogen bonds (with O...O distances ranging from 2.537(2) to 2.635(3) Å, graph set descriptor $R_{2}^{2}$(8)), and these pairs create hydrogen-bonded columns with water molecules (via O-H…O hydrogen bonds) along the *a*-axis. The resulting tubular crystal packing with phenyl rings oriented towards cavity axes is shown in Figure 5. Each hydrogen phosphate anion interacts with three water molecules and five cations, in addition to interacting with its neighbouring anion. The resulting hydrogen-bonded rings can be described by graph set descriptors $R_{1}^{2}$(4), $R_{2}^{1}$(6), $R_{2}^{2}$(8), $R_{3}^{2}$(8), and $R_{4}^{3}$(10).

**Figure 6:**
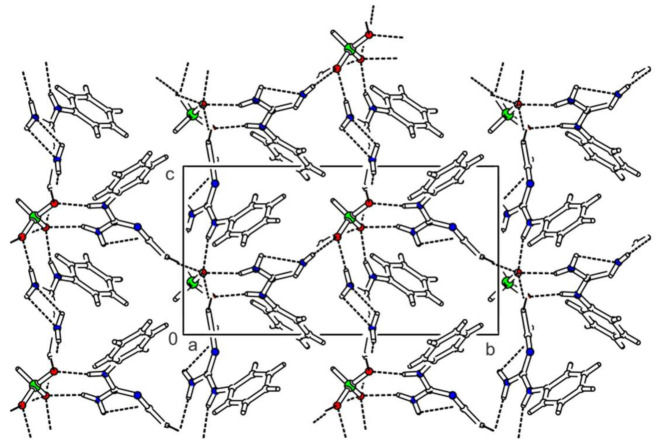
Packing scheme of **phbigua_2_HPO_3_** (view along the *a* axis direction); dashed lines indicate hydrogen bonds, solid lines indicate unit cell.

Bis(*N*-phenylbiguanidium(1+)) phosphite, **phbigua_2_HPO_3_**, crystallises in the monoclinic system (non-centrosymmetric space group *P*2_1_). Atom numbering is shown in Figure S5, Supplementary Materials. Its crystal structure is based on a 3D network consisting of alternating cations and anions interconnected through a system of N-H…O hydrogen bonds (see Figure 6). Each anion is hydrogen-bonded to six cations. Two of them are involved in simple N-H…O interactions (N15-H15A...O3 and N25-H25A...O1 with N…O distances of 2.775(2) and 2.783(3) Å, respectively). The remaining four are involved in pairs of hydrogen bonds (N11-H11…O2^a^ and N12-H12A…O1^a^, N14-H14B…O3^b^ and N15-H15B…O2^b^, N21-H21…O3^c^ and N22-H22A…O2^c^, N24-H24B…O1^b^ and N25-H25B…O2^b^) with N...O distances ranging from 2.821(2) to 3.156(3) Å and forming rings described by graph set descriptor $R_{2}^{2}$(8).

**Figure 7:**
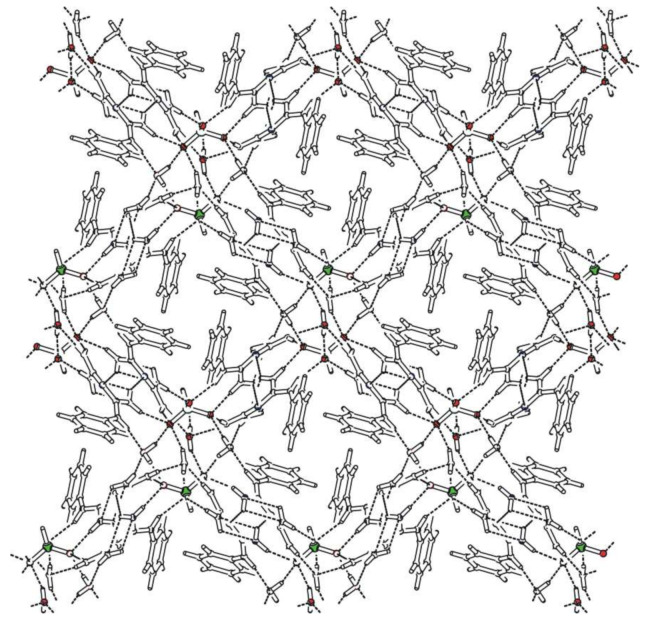
Packing scheme of **phbigua_2_HPO_3_2H_2_O** (view along the *b* axis direction); dashed lines indicate hydrogen bonds.

Bis(*N*-phenylbiguanidium(1+)) phosphite dihydrate, **phbigua_2_HPO_3_2H_2_O**, crystallises in the monoclinic system (space group *P*2_1_/*n*). Atom numbering is shown in Figure S6, Supplementary Materials. The cations form chains along the *b* axis through intermolecular N-H...N hydrogen bonds (i.e., N12-H12B…N13^a^ and N22-H22B…N23^d^ with N...N distances of 3.169(2) and 3.172(2) Å, respectively), which can be described by graph set descriptor C(4). Pairs of anions form a cluster with four water molecules (see Figure S7, Supplementary Materials) interconnected by O-H…O hydrogen bonds with O…O distances ranging from 2.685(1) to 2.812(1) Å. The hydrogen-bonded rings that are formed in this structure correspond to graph set descriptor $R_{2}^{3}$(10). These clusters are arranged along the *b*-axis, and the resulting tubular crystal packing is shown in Figure 7. Each anion is hydrogen-bonded to four water molecules and five cations (via N-H...O hydrogen bonds with N…O distances ranging from 2.784(2) to 2.960(2) Å), forming patterns described by graph set descriptors $R_{2}^{2}$(8) and $R_{3}^{2}$(8).

From a crystal engineering standpoint, the anions that are present in the structures can be divided into several sub-groups, according to different criteria. Considering their hydrogen bonding potential, we can distinguish strict H-bond acceptors (nitrates, perchlorates, sulfates, and phosphites) and mixed H-bond donors/acceptors (hydrogen carbonates and hydrogen phosphates). By considering only general symmetry (i.e., disregarding the position of their hydrogen atoms), we can differentiate planar (nitrates and hydrogen carbonates), trigonal pyramidal (hydrogen phosphates and phosphites), and tetrahedral (perchlorates and sulfates) anions.

Surprisingly, these criteria have only a limited effect on the final crystal packing. Planar anions present in **phbiguaNO_3_** and **phbiguaHCO_3_** crystals, as expected, allow the formation of layered structures with the phenyl ring oriented outwards from the double layers. Unexpectedly, the tetrahedral sulfate anions are involved in a very similar crystal packing in the non-centrosymmetric **phbigua_2_SO_4_**.

The trigonal pyramidal hydrogen phosphates and phosphites are involved in tubular structures of **phbigua_2_HPO_4_1.5H_2_O** and **phbigua_2_HPO_3_2H_2_O**, with their phenyl rings oriented towards the axes of the cavities. In addition, the presence of water molecules likely contributes to the formation of this type of crystal packing.

The crystal structure of **phbiguaClO_4_**, containing tetrahedral perchlorate anions, can be considered, to some extent, a crossover between a layered structure (with layers interconnected through intermolecular H-bonds forming centrosymmetric pairs of cations) and the 3D crystal packing that is observed in non-centrosymmetric **phbigua_2_HPO_3_**, containing pyramidal phosphite anions.

The conformation of *N*-phenylbiguanidium(1+) cations in the crystal structures is further stabilised by one (in **phbigua_2_SO_4_** and in **phbigua_2_HPO_3_**—one of the cations) or two N-H...N intramolecular hydrogen bonds (see Tables S3–S9, Supplementary Materials) described by graph set descriptor S(6). A very similar situation was also observed in the previously studied biguanide salts [24,29,32,33].

Most of these structures also contain at least one weak intramolecular C-H…N interaction (except for **phbiguaClO_4_**), which involves a phenyl ring C-H group and the non-protonated nitrogen N3 (or NY3, where Y = 1–8)—see Tables S3–S9, Supplementary Materials. Moreover, weak intermolecular interactions, more specifically C-H...N and C-H...O interactions, occur in the structures of **phbigua_2_HPO_3_** and **phbigua_2_HPO_4_1.5H_2_O**, respectively.

All aforementioned crystal structures of *N*-phenylbiguanidium(1+) salts contain an extensive network of hydrogen bonds involving nitrogen atoms. These interactions significantly affect the geometry of the cations. Cation conformation can be discussed based on two main parameters: (a) the interplanar angle of two plains of “guanidine” sub-skeletons forming the biguanide part of the cation and (b) the interplanar angle between the phenyl ring plane and the neighbouring “guanidine” plane.

The values of interplanar angles in the biguanide part of the cations range from 36 to 50°. To compare the values determined in previously studied biguanide and *N*-phenylbiguanide salts, see references [24,29,30,32,33]. The values of the interplanar angles of the phenyl ring plane and neighbouring “guanidine” plane range from 15 to 71°. Monitoring these conformation parameters has proved crucial for explaining the isostructural phase transformation mechanism of bis(*N*-phenylbiguanidium(1+)) oxalate [30].

### 2.2. Vibrational Spectra

**Figure 8:**
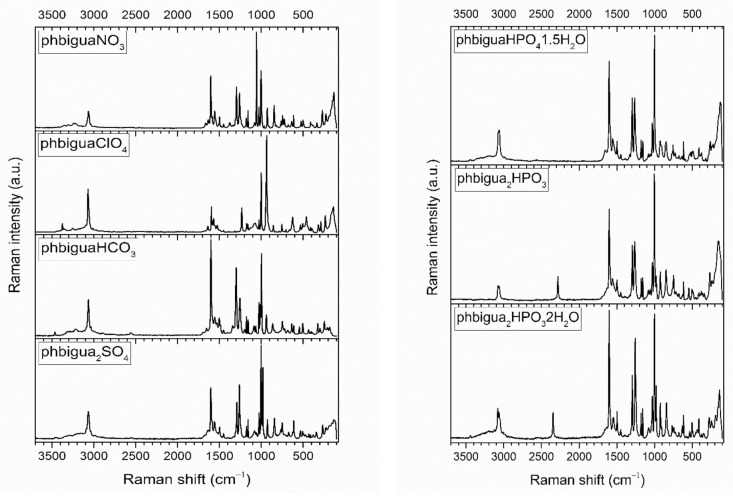
FT Raman spectra of *N*-phenylbiguanidium(1+) salts.

**Figure 9:**
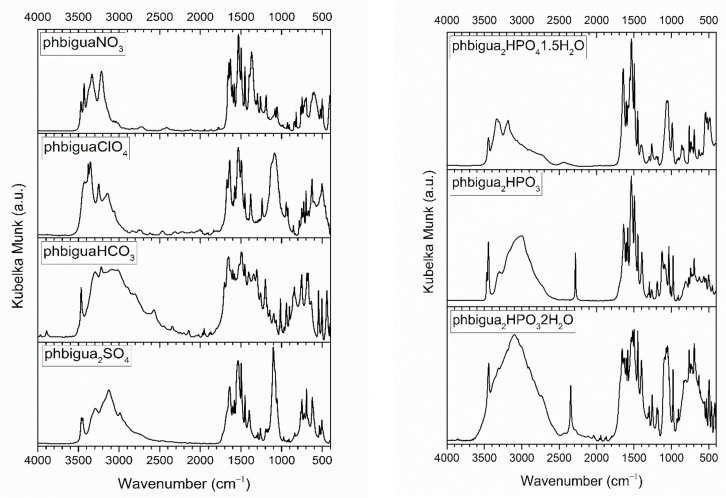
DRIFTS spectra of *N*-phenylbiguanidium(1+) salts.

Vibrational spectra of the inorganic salts of *N*-phenylbiguanidium(1+) are presented in Figure 8 and Figure 9, and the list of the recorded maxima is provided in Supplementary Materials (see Listing S1, Supplementary Materials). The IR spectra are dominated by structured, strong-to-medium intensity bands (3500–2500 cm^−1^ region) of stretching modes of N-H groups, mostly involved in N-H…O interactions. These bands are overlapping with similar stretching modes of O-H groups involved in O-H…N and O-H…O interactions in **phbiguaHCO_3_**, **phbigua_2_HPO_4_1.5H_2_O**, and **phbigua_2_HPO_3_2H_2_O**.

The intense bands that are recorded at 1375, 1122, 1400–1300, 1101, and 1045 cm^−1^ in the IR spectra of **phbiguaNO_3_**, **phbiguaClO_4_**, **phbiguaHCO_3_**, **phbigua_2_SO_4_**, and **phbigua_2_HPO_4_1.5H_2_O**, respectively, are typical manifestations of antisymmetric stretching modes of NO_3_^−^, ClO_4_^−^, HCO_3_^−^, SO_4_^2−^, and HPO_4_^2−^ anions. The presence of HPO_3_^2−^ anions is reflected by the bands of P-H stretching modes that are recorded at 2283 and 2347 cm^−1^ in both IR and Raman spectra of **phbigua_2_HPO_3_** and **phbigua_2_HPO_3_2H_2_O**, respectively. The position of these bands highlights slight differences in bonding conditions within these anions associated with shorter P-H bonds in **phbigua_2_HPO_3_2H_2_O** than in **phbigua_2_HPO_3_** (see Tables S8 and S9, Supplementary Materials).

Raman spectra of all seven salts contain bands of C-H stretching modes of an aromatic ring (3080–3060 cm^−1^ region). The intensity and shape of these bands can be correlated with the involvement of phenyl rings in C-H...X (X = N or O) hydrogen bonds (see Figure 8 and Tables S3–S9, Supplementary Materials). Strong symmetric bands were observed in **phbiguaClO_4_** and **phbiguaHCO_3_** with no or only single, weak intramolecular C-H...N interactions, respectively. The formation of slightly stronger intramolecular and/or intermolecular C-H...N or intermolecular C-H...O interactions is reflected by medium-intensity asymmetric doublets (**phbigua_2_SO_4_**, **phbiguaNO_3_**, **phbigua_2_HPO_3_**, and **phbigua_2_HPO_4_1.5H_2_O)** or even by the quadruplet (**phbigua_2_HPO_3_2H_2_O**). Other intense bands typical for phenyl groups were recorded at ~1600 cm^−1^ and ~1000 cm^−1^, corresponding to stretching C-C vibrations and “breathing” modes of phenyl rings, respectively. The sharp and very strong bands at 1056, 935, and 980 cm^−1^ in the Raman spectra of **phbiguaNO_3_**, **phbiguaClO_4_**, and **phbigua_2_SO_4_** can be assigned to symmetric stretching modes of NO_3_^−^, ClO_4_^−^, and SO_4_^2−^ anions, respectively.

The solid-state vibrational modes of **phbigua_2_SO_4_** and **phbigua_2_HPO_3_** crystals were devised (see Figures S8–S11, Supplementary Materials) within the computational characterisation of these non-centrosymmetric materials. In addition to the standard B3LYP functional, the advanced CAM-B3LYP functional (incorporating additional long-range corrections) was used to improve the agreement between computed vibrational modes and the recorded spectra of **phbigua_2_SO_4_**. The latter approach was particularly successful in improving the frequencies of the calculated modes (mainly of those involving contributions from the antisymmetric stretching vibrations of SO_4_^2−^ anions) in the 1150–1000 cm^−1^ region of the IR spectrum (see Figure S8, Supplementary Materials) and the intensities calculated below 950 cm^−1^ in the Raman spectrum (see Figure S9, Supplementary Materials).

These preliminary conclusions about recorded spectra are based on the literature on vibrational manifestations of inorganic anions [38] and on previous spectroscopic studies focused on bis(*N*-phenylbiguanidium(1+)) oxalate polymorphs, including *N*-phenylbiguanidium(1+) chloride [30]. The spectra of *N*-phenylbiguanide inorganic salts will be assigned in detail in a following paper.

### 2.3. Thermal Behaviour

To evaluate the thermal stability of the materials prepared in this study, DSC measurements were performed for all compounds in the temperature range from 93 K to a temperature close to their melting points. No thermal anomalies were recorded in the low-temperature region, and crystals of **phbiguaNO_3_** (m.p. 483 K), **phbiguaClO_4_** (m.p. 433 K), **phbiguaHCO_3_** (m.p. 401 K), **phbigua_2_SO_4_** (m.p. 483 K), **phbigua_2_HPO_4_1.5H_2_O** (m.p. 470 K), **phbigua_2_HPO_3_** (m.p. 451 K), and **phbigua_2_HPO_3_2H_2_O** (m.p. 393 K) were stable in air up to their melting points, which are listed in parentheses.

### 2.4. Linear and Nonlinear Optical Properties of **phbigua_2_SO_4_** and **phbigua_2_HPO_3_**

Based on the results from crystal structure determination and solid-state DFT computations, the non-centrosymmetric crystals of **phbigua_2_SO_4_** and **phbigua_2_HPO_3_** are optically anisotropic biaxial negative and biaxial positive materials, respectively. The values of the calculated refractive indices and static (λ = ∞) nonlinear susceptibility tensor *χ*^(2)^ components are presented in Table 3. The results indicate that **phbigua_2_SO_4_** crystals have larger birefringence than **phbigua_2_HPO_3_** crystals. The refractive indices of **phbigua_2_SO_4_** assessed using the CAM-B3LYP functional were systematically lower than those determined using the B3LYP functional. The results of the main *χ*^(2)^ component values of **phbigua_2_SO_4_** were, nevertheless, quite similar when comparing the functionals that were used in this study (except for the opposite sign of the of $\chi_{\mathrm{yyy}}^{\left(2\right)}$ component).

**Figure 10:**
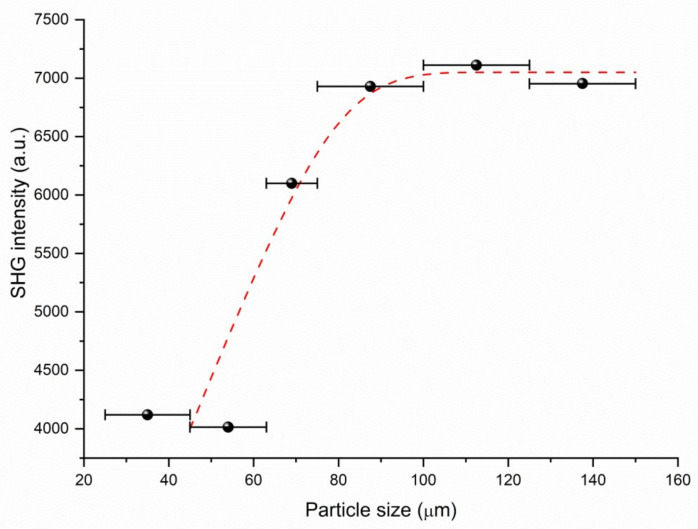
Phase-matching curve (i.e., particle size vs. SHG intensity) of **phbigua_2_SO_4_** (800 nm fundamental wavelength); the red curve drawn is to guide the eye and is not fitted to the data. Black horizontal segments represent particle size intervals.

The SHG measurements of powdered samples were performed for **phbigua_2_SO_4_** and **phbigua_2_HPO_3_** crystals, using powdered KDP as the reference material. These measurements yielded a relative SHG efficiency $d_{phbigua2SO4}$ = 5.57 *d***_KDP_** and $d_{phbigua2HPO3}$ = 1.60 *d***_KDP_** at 800 nm laser irradiation. The value of SHG efficiency determined for **phbigua_2_HPO_3_** is slightly underestimated due to the presence of approximately 5% (as determined by powder XRD) of the centrosymmetric **phbigua_2_HPO_3_2H_2_O** phase. The phase-matchability of the more promising **phbigua_2_SO_4_** was studied by particle-size-dependent measurements with an 800 nm laser line (see Figure 10). The results clearly confirm the possibility of SHG phase matching for this salt. The small discrepancy between the two smallest fractions can be explained by problems in preparing powders with isometric particles and effective control of the particle size in the family of soft molecular crystals, which are quite common (e.g., [22,39,40]).

For further optical characterisation of **phbigua_2_SO_4_**, UV–Vis spectra of its powdered sample were recorded (see Figure S12, Supplementary Materials), confirming the wide transparency range of this material, at least from the Vis region to 320 nm.

We have also preliminarily determined the second-order nonlinear optical tensor [$d_{ijk}^{SHG}$] of the **phbigua_2_SO_4_** crystal using the Maker-fringe method [41] at 1064 nm laser irradiation. The tensor of monoclinic **phbigua_2_SO_4_** (space group *C*2) has eight independent components and in the contracted form (Voigt’s notation using *d*_mn_ components), it reads as [42]:

$$\left(\begin{array}{ccc} 0\;\;\; & 0\;\;\; & 0\;\;\;\;\;\\ d_{21} & d_{22} & d_{23\;}\\ 0\;\;\; & 0\;\;\; & 0\;\;\;\;\; \end{array}\;\begin{array}{ccc} d_{14} & 0\;\;\; & d_{16}\\ 0\;\;\; & d_{25} & 0\;\;\;\\ d_{34} & 0\;\;\; & d_{36} \end{array}\right)$$

Unfortunately, accurate experimentally determined values of refractive indices remain unknown, and the size of the bulk single crystals grown was insufficient for their precise determination. Therefore, we were only able to estimate the magnitudes of *d*_mn_s. The results indicate that the component *d*_21_ is larger than the component *d*_36_ in reference KDP [43].

## 3. Materials and Methods

### 3.1. Syntheses

The aqueous solutions containing *N*-phenylbiguanide (98%, Sigma-Aldrich, St. Louis, MO, USA) and 2 mol L^−1^ solution of inorganic acid—i.e., nitric acid (68%, p.a., Lachema, Brno, Czech Republic), perchloric acid (70%, p.a., Lachema, Brno, Czech Republic), sulphuric acid (96%, p.a., Lachema, Brno, Czech Republic), phosphoric acid (purum, Lachema, Brno, Czech Republic), and phosphorous acid (p.a., Fluka, Seelze, Germany)—were prepared in molar ratios varying from 3:1 to 1:2 (base: acid) and left to crystallise spontaneously at room temperature. Crystals of **phbiguaNO_3_** and **phbiguaClO_4_** were isolated from systems based on a 1:1 molar ratio, and crystals of **phbigua_2_SO_4_**, **phbigua_2_HPO_4_1.5H_2_O** and **phbigua_2_HPO_3_2H_2_O** were formed in systems containing a 2:1 molar ratio. Crystals of **phbigua_2_HPO_3_** were prepared by recrystallisation (3 mL MeOH) of the precipitate formed in the reaction of 0.3 g of *N*-phenylbiguanide diluted in 6 mL of distilled H_2_O with 0.3 mL of a 2 mol L^−1^ solution of phosphorous acid. Crystals of **phbiguaHCO_3_** were prepared by slow evaporation of the aqueous solution (90 mL) containing 1.07 g of *N*-phenylbiguanide saturated with gaseous CO_2_ (30 min; pH of the solution decreased from ~10 to ~6).

### 3.2. Methods

The collection of X-ray diffraction data of suitable single crystals was performed on the following experimental devices: KappaCCD (Bruker Nonius, Billerica, MA, USA) diffractometer (MoK*_α_* radiation, graphite monochromator, COLLECT [44], and DENZO [45] software) for samples **phbiguaNO_3_**, **phbiguaClO_4_**, **phbiguaHCO_3_**, **phbigua_2_SO_4_**, **phbigua_2_HPO_4_1.5H_2_O**, and **phbigua_2_HPO_3_2H_2_O**; KM4 CCD (Oxford Diffraction Ltd., Abingdon, UK) diffractometer (CrysAlis CCD [46] software) for **phbigua_2_HPO_3_2H_2_O** and D8 VENTURE Kappa Duo (Bruker, Billerica, MA, USA) diffractometer with PHOTON III CMOS detector and IµS micro-source with MoK*_α_* radiation and SAINT V8.40B [47] software for **phbigua_2_HPO_3_.** The temperature of the crystal samples was controlled by a liquid nitrogen Cryostream Cooler (Oxford Cryosystems, Oxford, UK). The phase problem was solved by direct methods (with corresponding diffractometer software [48,49,50]) and the non-hydrogen atoms were refined anisotropically, using the full-matrix least-squares procedure (appropriate version of SHELX software [50,51]). Hydrogen atoms attached to carbon atoms were calculated in a geometrically idealised position with Csp^2^—H = 0.93 Å, and constrained to ride on their parent atoms, with *U_iso_*(H) = 1.5*U_eq_*(C). The positions of the hydrogen atoms attached to the oxygen and nitrogen atoms were localised on difference Fourier maps and were fixed during refinement using rigid body approximation with assigned displacement parameters equal to 1.2*U_iso_* (pivot atom). A hydrogen atom attached to a phosphorus atom was localised on a difference Fourier map and refined isotropically.

The determination of absolute structure of the non-centrosymmetric crystals was based on anomalous-dispersion effects of either the phosphorus or the sulphur atoms; the resulting chirality parameters [52] were 0.01(3) and −0.01(2) for **phbigua_2_HPO_3_** and **phbigua_2_SO_4_**, respectively.

The basic crystallographic data, measurement, and refinement details are summarised in Tables S1 and S2, Supplementary Materials. Selected bond lengths and angles, including those of hydrogen bonds, are presented in Tables S3–S9, Supplementary Materials. Crystallographic data for **phbiguaNO_3_**, **phbiguaClO_4_**, **phbiguaHCO_3_**, **phbigua_2_SO_4_**, **phbigua_2_HPO_4_1.5H_2_O**, **phbigua_2_HPO_3_**, and **phbigua_2_HPO_3_2H_2_O** were deposited at the Cambridge Crystallographic Data Centre as supplementary publications CCDC 789164, CCDC 789163, CCDC 880720, CCDC 741765, CCDC 789162, CCDC 2063178, and CCDC 789161, respectively. A copy of the data is available free of charge from CCDC, 12 Union Road, Cambridge CG21, EZ, UK (fax: +44 1223 336033; e-mail: deposit@ccdc.cam.ac.uk).

Powder X-ray diffraction patterns of prepared polycrystalline products (see Tables S10–S16, Supplementary Materials) were collected at room temperature using a Philips/PANalytical (Royston, UK) X’pert PRO MPD diffractometer (Bragg–Brentano geometry, ultrafast X’Celerator detector, and Cu K*α* radiation, λ = 1.5418 Å). Theoretical diffraction patterns for confirmation of phase purity of prepared products were calculated using the FullProf software [53].

Infrared spectra of powdered samples were recorded by DRIFTS technique (samples mixed with KBr at ~1:50 ratio and grinded in agate mortar) on a Thermo Fisher Scientific (Madison, WI, USA) Nicolet 6700 FTIR spectrometer in the 400–4000 cm^−1^ region (2 cm^−1^ resolution, KBr beamsplitter, Happ–Genzel apodization). Raman spectra of powdered samples were recorded on a Nicolet 6700 FTIR spectrometer equipped with a Thermo Fisher Scientific (Madison, WI, USA) Nicolet Nexus FT Raman module (2 cm^−1^ resolution, Happ–Genzel apodization, 1064 nm Nd:YVO_4_ laser excitation, ~400 mW power at the sample) in the 100–3700 cm^−1^ region. Raman spectra of microcrystalline samples were also collected on a dispersive confocal Raman microscope MonoVista CRS+ (Spectroscopy & Imaging GmbH, Warstein, Germany) interfaced to an Olympus microscope (objectives 20x and 50x) using a 785 nm diode excitation laser (10 mW laser power, 40–3800 cm^−1^ spectral range, 300 lines/mm grating). The spectrometer was wavelength- and intensity-calibrated using a software-controlled auto-alignment and calibration procedure with mercury and Ne-Ar lamps.

The UV–Vis spectra of **phbigua_2_SO_4_** powdered samples were recorded using an Agilent Technologies (Santa Clara, CA, USA) Cary Series 4000 UV–Vis spectrometer equipped with internal DRA-900 accessory (diffuse reflectance with integrating sphere) in the 190–850 nm spectral region with a 0.5 nm spectral resolution.

DSC measurements were carried out on Perkin Elmer (Waltham, MA, USA) Pyris Diamond DSC and DSC 7 instruments in the 93–473 K temperature region (20 mL/min nitrogen or helium flow above and below 298 K, respectively). A heating rate of 10 K/min was selected to measure approximately 20 mg of the finely ground sample placed in hermetically sealed aluminium pans.

SHG measurements of powdered **phbigua_2_HPO_3_** and **phbigua_2_SO_4_** were performed using the modified Kurtz–Perry method [54]. The samples were irradiated with 90 fs laser pulses generated at an 82 MHz repetition rate by a Spectra Physics (Santa Clara, CA, USA) Tsunami Ti:sapphire laser at 800 nm. For quantitative determination of SHG efficiency, the intensity of the back-scattered laser light generated in the sample at 400 nm was measured on a grating spectrograph with a diode array (InstaSpect II, Oriel Newport Corporation, Irvine, CA, USA), and the signal was compared with that produced by a potassium dihydrogen phosphate (KDP) standard. The initial experiments were performed on a powdered sample (75–150 μm particle size) loaded into a 5 mm glass cell using a mechanical vibrator. The measurements were repeated on different areas of the same sample, and the results were averaged. This experimental procedure was employed to minimise signal fluctuations induced by sample packing. Lastly, the measurements of **phbigua_2_SO_4_** were performed also with size-fractioned samples (particle size: 25–45, 45–63, 63–75, 75–100, 100–125, and 125–150 μm).

The SHG study focused on the determination of particular *d*_mn_ coefficients of the second-order NLO tensor that were performed on cuts of **phbigua_2_SO_4_** single crystals using the standard Maker-fringe method [41]; with a BMI (Evry, France) Q-switched Nd-YAG laser (1064 nm, ~6 ns pulses of less than 0.1 mJ at 20 Hz) as a light source. For details of the applied measurement strategy (monoclinic crystals of point group 2), see e.g., [31]. Single crystals were grown by isothermal crystallisation of aqueous solution at 296 K in a dark room for several months.

Solid-state DFT studies of **phbigua_2_SO_4_** and **phbigua_2_HPO_3_** were carried out using the CRYSTAL17 program (version 1.0.2.) [55]. Primary computations employed the B3LYP functional; the 6-31+G(d,p) basis set was used for all oxygen and the 6-31G(d) basis set for all other atoms. DFT integrations utilised the large integration grid implemented in the program. The Pack–Monkhorst and Gilat nets used for sampling the Brillouin zone consisted each of 8 points. Derivatives required to compute IR and Raman spectra were obtained by the coupled-perturbed Kohn–Sham analytical approach [56,57]. Unit cell parameters were not optimised. The starting atomic coordinates were those obtained from diffraction experiments, which were subjected to optimisation that used tightened convergence criteria. Spectral and optical properties were subsequently computed on the stationary-point geometries obtained. Theoretical Raman spectra were corrected empirically for experimental wavelength 1064 nm and temperature 293 K. Vibrational frequencies obtained from computations were scaled uniformly by an empirical factor of 0.97. To improve the fit of computed vibrational frequencies and recorded spectra of **phbigua_2_SO_4_**, the CAM-B3LYP functional and the triple-zeta POB-TZVP basis set [58] for all atoms was also used. The Pack–Monkhorst and Gilat nets used for sampling the Brillouin zone in this case consisted each of 12 points. Tolerance criteria for coulomb and exchange sums were tightened by using the respective values 7, 7, 7, 9, and 30 for the TOLINTEG input keyword. Vibrational frequencies obtained from computations were scaled uniformly by an empirical factor of 0.96.

## 4. Conclusions

Seven novel salts were prepared and characterised in studied systems containing *N*-phenylbiguanide and selected inorganic oxyacids. The involved inorganic anions differ in their symmetry and in the role that they play in hydrogen bond networks. The crystal packing of these salts ranges from layered (**phbiguaNO_3_**, **phbiguaHCO_3_**, and **phbigua_2_SO_4_**) to tubular (**phbigua_2_HPO_4_1.5H_2_O** and **phbigua_2_HPO_3_2H_2_O**) structures, which are enabled not only by the suitable symmetry of their anions but also by the involvement of water molecules in the H-bonding patterns. Two salts—**phbigua_2_SO_4_** and **phbigua_2_HPO_3_**—met our expectations of a non-centrosymmetric crystal arrangement, necessary for observing *χ*^(2)^ NLO effects.

The results from the recorded vibrational spectra match the results of crystal structure determination, i.e., crystals based on hydrogen-bonded networks containing *N*-phenylbiguanidium(1+) cations and inorganic anions (eventually water molecules). The formation of various hydrogen bonds is reflected in vibrational spectra by the presence of strong wide bands (IR spectra) of stretching vibrations of N-H and O-H groups involved in these bonds, by the different positions of P-H stretching modes in **phbigua_2_HPO_3_2H_2_O** and **phbigua_2_HPO_3_** (both spectra) and by the shape of the bands of C-H stretching vibrations in **phbigua_2_SO_4_**, **phbiguaNO_3_**, **phbigua_2_HPO_3_**, **phbigua_2_HPO_4_1.5H_2_O**, and **phbigua_2_HPO_3_2H_2_O** (Raman spectra).

Powder SHG measurements of **phbigua_2_SO_4_** coupled with preliminary single-crystal optical measurements confirmed that this compound especially is a promising (SHG efficient, phase-matchable, transparent down to 320 nm, and thermally stable to 483 K) novel material for NLO. In addition to favourable *χ*^(2)^ NLO properties, both non-centrosymmetric salts could also be considered as potential SRS-active materials for Raman laser converters. Especially intense sharp Raman bands of sulfate symmetric stretching vibration of **phbigua_2_SO_4_** and phosphite stretching P-H vibration of **phbigua_2_HPO_3_** could, similarly as in relative guanidine-based compounds [10,11], act as SRS-promoting vibrational modes. Unfortunately, problems related to bulk single-crystals growth complicate broader technical applications of **phbigua_2_SO_4_** crystals.

## Figures and Tables

**Table 1:** Basic crystallographic data of **phbiguaNO_3_**, **phbiguaClO_4_**, **phbiguaHCO_3_**, and **phbigua_2_SO_4_**.

Identification Code	phbiguaNO_3_	phbiguaClO_4_	phbiguaHCO_3_	phbigua_2_SO_4_
Crystal system	Monoclinic	Triclinic	Monoclinic	Monoclinic
Space group	*C*2/*c*	*P*-1	*P*2_1_/*c*	*C*2
*a* (Å)	18.9690 (5)	7.4100 (3)	9.9638 (3)	17.6320 (6)
*b* (Å)	6.1080 (2)	9.1199 (3)	7.1842 (2)	6.5130 (2)
*c* (Å)	20.3420 (7)	9.7919 (5)	16.1301 (5)	10.7200 (5)
*α* (°)	90	97.990 (3)	90	90
*β* (°)	98.870 (2)	110.418 (2)	94.553 (2)	118.706 (2)
*γ* (°)	90	96.388 (3)	90	90
V (Å^3^)	2328.7 (1)	604.96 (4)	1150.98 (6)	1079.75 (7)
*Z*	8	2	4	2
T (K)	293 (2)	293 (2)	150 (2)	293 (2)

**Table 2:** Basic crystallographic data of **phbigua_2_HPO_4_1.5H_2_O**, **phbigua_2_HPO_3_**, and **phbigua_2_HPO_3_2H_2_O**.

Identification Code	phbigua_2_HPO_4_1.5H_2_O	phbigua_2_HPO_3_	phbigua_2_HPO_3_2H_2_O
Crystal system	Triclinic	Monoclinic	Monoclinic
Space group	*P*-1	*P*2_1_	*P*2_1_/*n*
*a* (Å)	14.7620 (2)	6.3624 (2)	16.483 (5)
*b* (Å)	17.4790 (5)	17.4832 (6)	7.859 (1)
*c* (Å)	18.6460 (6)	9.8646 (3)	17.125 (3)
*α* (°)	91.143 (1)	90	90
*β* (°)	90.130 (2)	108.189 (1)	92.797 (17)
*γ* (°)	114.5310 (15)	90	90
V (Å^3^)	4375.7 (2)	1042.46 (6)	2215.6 (8)
*Z*	8	2	4
T (K)	150 (2)	150 (2)	150 (2)

**Table 3:** Calculated refractive indices and independent *χ*^(2)^ tensor components (A.U.) of **phbigua_2_SO_4_** and **phbigua_2_HPO_3_** crystals (λ = ∞).

	phbigua_2_SO_4_		phbigua_2_HPO_3_
	B3LYP	CAM-B3LYP	B3LYP
*n*_a_	1.460	1.439	1.543
*n*_b_	1.588	1.566	1.549
*n*_c_	1.594	1.574	1.610
$\chi_{\mathrm{xxx}}^{\left(2\right)}$	−1 × 10^−26^	1 × 10^−26^	6 × 10^−25^
$\chi_{\mathrm{xxy}}^{\left(2\right)}$	**−1.59**	**−1.12**	**−0.82**
$\chi_{\mathrm{xxz}}^{\left(2\right)}$	2 × 10^−26^	−2 × 10^−26^	4 × 10^−25^
$\chi_{\mathrm{xyy}}^{\left(2\right)}$	−2 × 10^−17^	−2 × 10^−17^	−3 × 10^−16^
$\chi_{\mathrm{xyz}}^{\left(2\right)}$	**0.83**	**0.56**	**−1.19**
$\chi_{\mathrm{xzz}}^{\left(2\right)}$	−1 × 10^−26^	2 × 10^−26^	1 × 10^−24^
$\chi_{\mathrm{yyy}}^{\left(2\right)}$	**−0.27**	**0.26**	**−0.53**
$\chi_{\mathrm{yyz}}^{\left(2\right)}$	2 × 10^−16^	1 × 10^−16^	−2 × 10^−16^
$\chi_{\mathrm{yzz}}^{\left(2\right)}$	**0.30**	**0.22**	**−0.08**
$\chi_{\mathrm{zzz}}^{\left(2\right)}$	0	0	0

*Note.* Main *χ*^(2)^ components are marked as bold numbers.

## Data Availability

The data presented in this study are available on request from the corresponding author.

## Supplementary Materials

The following are available online at https://www.mdpi.com/article/10.3390/ijms22168419/s1, Table S1: Basic crystallographic data and structure refinement details for phbiguaNO_3_, phbiguaClO_4_, phbigua_2_HPO_4_1.5H_2_O, and phbigua_2_HPO_3_2H_2_O. Table S2: Basic crystallographic data and structure refinement details for phbigua_2_HPO_3_, phbigua_2_SO_4_, and phbiguaHCO_3_. Table S3: Selected bond lengths [Å] and angles [°] for phbiguaNO_3_. Table S4: Selected bond lengths [Å] and angles [°] for phbiguaClO_4_. Table S5: Selected bond lengths [Å] and angles [°] for phbiguaHCO_3_. Table S6: Selected bond lengths [Å] and angles [°] for phbigua_2_SO_4_. Table S7: Selected bond lengths [Å] and angles [°] for phbigua_2_HPO_4_1.5H_2_O. Table S8: Selected bond lengths [Å] and angles [°] for phbigua_2_HPO_3_. Table S9: Selected bond lengths [Å] and angles [°] for phbigua_2_HPO_3_2H_2_O. Figure S1: Atom numbering of phbiguaNO_3_. Figure S2: Atom numbering of phbiguaClO_4_. **Figure S3**: Atom numbering of phbiguaHCO_3_. Figure S4: Atom numbering of phbigua_2_SO_4_. Figure S5: Atom numbering of phbigua_2_HPO_3_. Figure S6: Atom numbering of phbigua_2_HPO_3_2H_2_O. Figure S7: Detail of pairs of phosphite anions and water molecules inter-connected via hydrogen bonds in the crystal structure of phbigua_2_HPO_3_2H_2_O. Listing S1: List of recorded IR and Raman maxima (cm^−1^) of phbiguaNO_3_, phbiguaClO_4_, phbiguaHCO_3_, phbigua_2_SO_4_, phbigua_2_HPO_4_1.5H_2_O, phbigua_2_HPO_3_, and phbigua_2_HPO_3_2H_2_O. Figure S8: The comparison of calculated (CRYSTAL17 programme) vibrational modes using CAM-B3LYP (red lines) and B3LYP (green lines) functionals with recorded DRIFTS spectrum of phbigua_2_SO_4_. Figure S9: The comparison of calculated (CRYSTAL17 programme) vibrational modes using CAM-B3LYP (red lines) and B3LYP (green lines) functionals with recorded Raman spectrum of phbigua_2_SO_4_. Figure S10: The comparison of calculated (CRYSTAL17 programme) vibrational modes using B3LYP (green lines) functional with recorded DRIFTS spectrum of phbigua_2_HPO_3_. Figure S11: The comparison of calculated (CRYSTAL17 programme) vibrational modes using B3LYP (green lines) functional with recorded Raman spectrum of phbigua_2_HPO_3_. Figure S12: UV–Vis spectrum of powdered phbigua_2_SO_4_. Table S10: Experimental powder diffraction data for phbiguaNO_3_. Table S11: Experimental powder diffraction data for phbiguaClO_4_. Table S12: Experimental powder diffraction data for phbiguaHCO_3_. Table S13: Experimental powder diffraction data for phbigua_2_SO_4_. Table S14: Experimental powder diffraction data for phbigua_2_HPO_4_1.5H_2_O. Table S15: Experimental powder diffraction data for phbigua_2_HPO_3_. Table S16: Experimental powder diffraction data for phbigua_2_HPO_3_2H_2_O.
