# Supplementary material for: Inorganic Salts of N-phenylbiguanidium(1+)—Novel Family with Promising Representatives for Nonlinear Optics
Source: Int J Mol Sci. 2021 Aug 5;22(16):8419. doi: 10.3390/ijms22168419 (PMC8395133; doi:10.3390/ijms22168419)
Supplement: Supplementary file 1 [file ijms-22-08419-s001.zip › ijms-1295605-supplementary.pdf]

Supplementary Materials

for

Inorganic salts of *N*-phenylbiguanidium(1+) – novel family with promising representatives for nonlinear optics

Irena Matulková[1], Ivana Císařová [1], Michaela Fridrichová [1], Róbert Gyepes [2], Petr Němec [3], Jan Kroupa [4] and Ivan Němec [1,*]

[1] Department of Inorganic Chemistry, Faculty of Science, Charles University, Hlavova 8, Prague 2, 128 40 (Czech Republic); ivan.nemec@natur.cuni.cz
[2] Department of Molecular Electrochemistry and Catalysis, J. Heyrovský Institute of Physical Chemistry, Czech Academy of Sciences, Dolejškova 3, Prague 8, 182 23 (Czech Republic); robert.gyepes@jh-inst.cas.cz
[3] Department of Chemical Physics and Optics, Faculty of Mathematics and Physics, Charles University, Ke Karlovu 3, Prague 2, 121 16 (Czech Republic); nemec@karlov.mff.cuni.cz
[4] Department of Dielectrics, Institute of Physics, Czech Academy of Sciences, Na Slovance 2, Prague 8, 182 21 (Czech Republic); kroupa@fzu.cz

* Correspondence: ivan.nemec@natur.cuni.cz; Tel.: +420 221 951 247

Table S1. Basic crystallographic data and structure refinement details for **phbiguaNO3**, **phbiguaClO4**, **phbigua2HPO41.5H2O**, and **phbigua2HPO32H2O**.

Identification code	**phbiguaNO$_3$**	**phbiguaClO$_4$**	**phbigua$_2$HPO$_4$1.5H$_2$O**	**phbigua$_2$HPO$_3$2H$_2$O**
Empirical formula	$C_8 H_{12} N_6 O_3$	$C_8 H_{12} Cl N_5 O_4$	$C_{16} H_{28} N_{10} O_{5.5} P$	$C_{16} H_{29} N_{10} O_5 P$
Formula weight	240.24	277.68	479.45	472.46
Temperature	293(2) K	293(2) K	150(2) K	150(2) K
a	18.9690(5) Å	7.4100(3) Å	14.7620(2) Å	16.483(5) Å
b	6.1080(2) Å	9.1199(3) Å	17.4790(5) Å	7.8587(12) Å
c	20.3420(7) Å	9.7919(5) Å	18.6460(6) Å	17.125(3) Å
α	90 °	97.990(3) °	91.1430(11) °	90 °
β	98.8700(17) °	110.418(2) °	90.1300(19) °	92.797(17) °
γ	90 °	96.388(3) °	114.5310(15) °	90 °
Volume	2328.69(13) Å^3	604.96(4) Å^3	4375.7(2) Å^3	2215.6(8) Å^3
Z	8	2	8	4
Calculated density	1.370 Mg/m^3	1.524 Mg/m^3	1.456 Mg/m^3	1.416 Mg/m^3
Crystal system	Monoclinic	Triclinic	Triclinic	Monoclinic
Space group	$C2/c$	P-1	P-1	$P2_1/n$
Absorption coefficient	0.108 mm^{-1}	0.332 mm^{-1}	0.180 mm^{-1}	0.175 mm^{-1}
F(000)	1008	288	2024	1000
Crystal size	0.35 x 0.27 x 0.12 mm	0.5 x 0.2 x 0.15 mm	0.5 x 0.27 x 0.175 mm	0.329 x 0.22 x 0.12 mm
Diffractometer and radiation	Nonius Kappa CCD, Mo λ = 0.71073 Å			KM4 CCD, Mo λ =0.71069 Å
Scan technique	ω and φ scans to fill the Ewald sphere			
Completeness to θ	99.30%	99.30%	97.70 %	99.90%
Range of h, k and l	-23 → 24, -7 → 7, -26 → 26	-9 → 9, -11 → 11, -12 → 11	-19 →19, -22 → 22, -24 → 24	-20 → 20, -9 → 9, -21 → 21
θ range for data collection	3.51 to 27.50 °	2.89 to 27.47 °	1.28 to 27.49 °	3 to 26.5 °
Reflection collected/unique (R_{int})	22761 / 2648 (0.0209)	7394 / 2763 (0.0332)	62854 / 19632 (0.0531)	28790 / 4630 (0.0273)
No. of observed reflection	2054	2203	14602	3766
Criterion for observed reflection	$I > 2\sigma(I)$			
Absorption correction	none			
Function minimized	$\Sigma\ w(F_o^2 - F_c^2)^2$			
Parameters refined	154	164	1175	293
R; wR [$I>2s(I)$]	0.0473; 0.1210	0.0822; 0.2379	0.0474; 0.1080	0.0308; 0.0909
R; wR (all data)	0.0616; 0.1317	0.0963; 0.2560	0.0769; 0.1224	0.0385; 0.0935
Value of S	1.054	1.087	1.037	1.120
Max and min. heights in final $\Delta\rho$ map	0.173 and –0.190 e.Å^{-3}	1.130 and –0.697 e.Å^{-3}	0.506 and –0.462 e.Å^{-3}	0.437 and –0.340 e.Å^{-3}
Weighting scheme	$w = [\sigma^2(F_o^2) + aP^2 + bP]^{-1}$			
	$P = (F_o^2 + 2F_c^2)/3$			
	a = 0.0608	a = 0.1481	a = 0.0491	a = 0.0560
	b = 0.9527	b = 0.5740	b = 2.6699	b = 0.2924

Table S2. Basic crystallographic data and structure refinement details for **phbigua$_2$HPO$_3$**, **phbigua$_2$SO$_4$** and **phbiguaHCO$_3$**.

Identification code	**phbigua$_2$HPO$_3$**	**phbigua$_2$SO$_4$**	**phbiguaHCO$_3$**
Empirical formula	$C_{16}\,H_{25}\,N_{10}\,O_3\,P$	$C_{16}\,H_{24}\,N_{10}\,O_4\,S$	$C_9\,H_{13}\,N_5\,O_3$
Formula weight	436.43	452.51	239.24
Temperature	150(2) K	293(2) K	150(2) K
a	6.3624(2) Å	17.6320(6) Å	9.9638(3) Å
b	17.4832(6) Å	6.5130(2) Å	7.1842(2) Å
c	9.8646(3) Å	10.7200(5) Å	16.1301(5) Å
α	90 °	90 °	90 °
β	108.1890(10) °	118.7061(16) °	94.5525(16) °
γ	90 °	90 °	90 °
Volume	1042.46(6) Å^3	1079.75(7) Å^3	1150.98(6) Å^3
Z	2	2	4
Calculated density	1.390 Mg/m^3	1.392 Mg/m^3	1.381 Mg/m^3
Crystal system	Monoclinic	Monoclinic	Monoclinic
Space group	$P2_1$	$C2$	$P2_1/c$
Absorption coefficient	0.173 mm^{-1}	0.196 mm^{-1}	0.107 mm^{-1}
$F(000)$	460	476	504
Crystal size	0.325 x 0.273 x 0.174 mm	0.5 x 0.3 x 0.27 mm	0.4 x 0.4 x 0.3 mm
Diffractometer and radiation	Bruker D8 VENTURE Kappa Duo PHOTON III CMOS, Mo λ = 0.71073 Å	Nonius Kappa CCD, Mo λ = 0.71073 Å	
Scan technique	ω and φ scans to fill the Ewald sphere		
Completeness to θ	99.20%	98.20 %	99.80 %
Range of h, k and l	-8 → 8, -22 → 22, -10 → 12	-22 →22, -8 → 8, -13 → 13	-12 → 12, -9 → 9, -20 → 20
θ range for data collection	2.330 to 27.502 °	4.34 to 27.45 °	2.050 to 27.50 °
Reflection collected/unique (R_{int})	13672 / 4723 (0.0299)	7017 / 2443 (0.0230)	14499 / 2632 (0.0269)
No. of observed reflection	4675	2378	2118
Criterion for observed reflection	$I > 2\,\sigma(I)$		
Absorption correction	multi-scan	none	
Function minimized	$\Sigma\, w(F_o^2 - F_c^2)^2$		
Parameters refined	274	141	154
R; *wR* [$I>2s(I)$]	0.0254; 0.0666	0.0370; 0.1020	0.0498; 0.1087
R; *wR* (all data)	0.0256; 0.0670	0.0382; 0.1038	0.0385; 0.1018
Value of *S*	1.043	1.060	1.081
Max and min. heights in final $\Delta\rho$ map	0.210 and -0.195 e.Å^{-3}	0.249 and -0.274 e.Å^{-3}	0.210 and -0.236 e.Å^{-3}
Weighting scheme	$w = [\sigma^2(F_o^2) + aP^2 + bP]^{-1}$ $P = (F_o^2 + 2F_c^2)/3$		
	a = 0.0392	a = 0.0619	a = 0.0647
	b = 0.1575	b = 0.3414	b = 0.0986

Table S3. Selected bond lengths [Å] and angles [°] for **phbiguaNO$_3$**.

Bond/Angle	Value (Å/°)	Angle	Value (°)
N1 - C1	1.3501(18)	N3 - C1 - N2	125.48(13)
N1 - C3	1.4112(19)	N3 - C1 - N1	119.19(13)
N2 - C1	1.3311(19)	N2 - C1 - N1	115.25(12)
N3 - C1	1.3239(18)	N4 - C2 - N5	117.88(14)
N3 - C2	1.3333(18)	N4 - C2 - N3	117.57(14)
N4 - C2	1.3269(19)	N5 - C2 - N3	124.43(13)
N5 - C2	1.331(2)	C8 - C3 - C4	119.69(16)
C3 - C8	1.384(2)	C8 - C3 - N1	116.93(14)
C3 - C4	1.386(2)	C4 - C3 - N1	123.32(15)
C4 - C5	1.388(3)	C3 - C4 - C5	118.61(19)
C5 - C6	1.376(4)	C6 - C5 - C4	121.4(2)
C6 - C7	1.368(4)	C7 - C6 - C5	119.66(19)
C7 - C8	1.380(3)	C6 - C7 - C8	119.9(2)
N6 - O1	1.2335(16)	C7 - C8 - C3	120.74(18)
N6 - O2	1.2408(16)	O1 - N6 - O2	120.05(12)
N6 - O3	1.2406(16)	O1 - N6 - O3	119.37(13)
C1 - N1 - C3	128.23(12)	O2 - N6 - O3	120.57(12)
C1 - N3 - C2	121.84(13)		

Hydrogen bonds

D-H...A	d(D-H)	d(H...A)	d(D...A)	<(DHA)
N1 - H1...O2[a]	0.929	2.04	2.9627(17)	173
N2 - H2A...O3[b]	0.8755	2.18	2.9469(19)	145
N2 - H2A…N5	0.8755	2.52	2.8888(18)	107
N2 - H2B...O3[a]	0.9246	2.01	2.9063(16)	164
N4 - H4A...O2[c]	0.925	2.11	3.0314(17)	171
N4 - H4B...O1[d]	0.9636	1.97	2.9263(17)	175
N5 - H5A...N2	0.9002	2.46	2.8888(18)	110
N5 - H5B...O1[c]	0.8979	2.01	2.8927(18)	166
C4 - H4…N3	0.9300	2.48	2.922(2)	109

Note. Equivalent positions: (a) 1/2-x, 3/2-y, -z; (b) x, 1+y, z; (c) 1-x, 2-y, -z; (d) 1-x, 1-y, -z

Table S4. Selected bond lengths [Å] and angles [°] for **phbiguaClO₄**.

Bond/Angle	Value (Å/°)	Angle	Value (°)
C1 - N2	1.329(4)	N5 - C2 - N3	118.0(3)
C1 - N3	1.333(4)	N4 - C2 - N3	123.4(3)
C1 - N1	1.336(4)	C8 - C3 - C4	120.4(3)
C2 - N5	1.321(4)	C8 - C3 - N1	119.6(3)
C2 - N4	1.332(5)	C4 - C3 - N1	120.0(3)
C2 - N3	1.343(4)	C3 - C4 - C5	119.3(4)
C3 - C8	1.376(6)	C6 - C5 - C4	120.5(4)
C3 - C4	1.388(5)	C5 - C6 - C7	119.8(4)
C3 - N1	1.431(4)	C6 - C7 - C8	120.6(4)
C4 - C5	1.388(5)	C3 - C8 - C7	119.4(4)
C5 - C6	1.366(6)	C1 - N1 - H1	117.8
C6 - C7	1.379(7)	C3 - N1 - H1	116.2
C7 - C8	1.385(6)	C1 - N2 - H2A	118.0
N1 - H1	0.8881	C1 - N2 - H2B	121.5
N2 - H2A	0.8988	H2A - N2 - H2B	118.9
N2 - H2B	0.9341	C1 - N3 - C2	122.3(3)
N4 - H4A	0.9137	C2 - N4 - H4A	114.4
N4 - H4B	0.9712	C2 - N4 - H4B	117.2
N5 - H5A	0.9056	H4A - N4 - H4B	127.8
N5 - H5B	0.9666	C2 - N5 - H5A	121.6
Cl1 - O1	1.322(5)	C2 - N5 - H5B	121.2
Cl1 - O2	1.397(4)	H5A - N5 - H5B	115.3
Cl1 - O3	1.400(4)	O1 - Cl1 - O2	114.2(4)
Cl1 - O4	1.407(6)	O1 - Cl1 - O3	110.3(5)
N2 - C1 - N3	124.8(3)	O2 - Cl1 - O3	114.5(3)
N2 - C1 - N1	118.3(3)	O1 - Cl1 - O4	106.2(7)
N3 - C1 - N1	116.8(3)	O2 - Cl1 - O4	109.0(3)
N5 - C2 - N4	118.5(3)	O3 - Cl1 - O4	101.5(4)

Hydrogen bonds

D-H...A	d(D-H)	d(H...A)	d(D...A)	<(DHA)
N2 - H2A...O1	0.90	2.01	2.850(6)	156
N2 - H2B...O3[a]	0.93	2.45	3.079(6)	125
N2 - H2B...O4[a]	0.93	2.46	3.181(6)	135
N2 - H2A…N4	0.90	2.55	2.918(5)	105
N4 - H4A…O3[illegible]	0.91	2.14	3.046(6)	169
N4 - H4B...O2[c]	0.97	1.99	2.962(6)	179
N4 - H4A…N2	0.91	2.57	2.918(5)	103
N5 - H5A...O4[c]	0.91	2.14	3.025(6)	166
N5 - H5B...N3[d]	0.97	2.04	3.006(4)	176

Note. Equivalent positions: (a) 1-x, -y, 1-z; (b) -1+x, y, z; (c) 1-x, 1-y, 1-z; (d) 1-x, 1-y, 2-z

Table S5. Selected bond lengths [Å] and angles [°] for **phbiguaHCO$_3$**.

**Bond/Angle**	**Value (Å/°)**	**Angle**	**Value (°)**	
N1 - C1	1.3473(14)	N2 - C1 - N3	124.39(10)	
N1 - C3	1.4133(14)	N2 - C1 - N1	114.76(10)	
N2 - C1	1.3369(15)	N3 - C1 - N1	120.71(10)	
N3 - C1	1.3409(14)	N5 - C2 - N4	118.29(10)	
N3 - C2	1.3605(14)	N5 - C2 - N3	117.85(10)	
N4 - C2	1.3287(14)	N4 - C2 - N3	123.74(10)	
N5 - C2	1.3206(15)	C4 - C3 - C8	119.44(11)	
C3 - C4	1.3932(17)	C4 - C3 - N1	124.32(10)	
C3 - C8	1.3950(17)	C8 - C3 - N1	116.13(10)	
C4 - C5	1.3896(18)	C5 - C4 - C3	119.27(11)	
C5 - C6	1.382(2)	C6 - C5 - C4	121.26(13)	
C6 - C7	1.381(2)	C7 - C6 - C5	119.56(12)	
C7 - C8	1.3901(17)	C6 - C7 - C8	120.04(12)	
C9 - O3	1.2421(13)	C7 - C8 - C3	120.43(12)	
C9 - O2	1.2607(13)	O3 - C9 - O2	126.30(10)	
C9 - O1	1.3521(13)	O3 - C9 - O1	115.77(10)	
C1 - N1 - C3	130.32(10)	O2 - C9 - O1	117.91(9)	
C1 - N3 - C2	119.86(9)	C9 - O1 - H1O	113.8	
**Hydrogen bonds**				
**D-H...A**	**d(D-H)**	**d(H...A)**	**d(D...A)**	**<(DHA)**
O1 - H1O…N3[a]	0.8991	1.91	2.7943(12)	168
N1 - H1...O2	0.9413	1.94	2.8782(13)	174
N2 - H2A...N4	0.9166	2.50	2.8487(14)	103
N2 - H2A...O2[b]	0.9166	2.00	2.8728(13)	160
N2 - H2B...O3	0.9359	1.88	2.8062(13)	169
N4 - H4A...O1[c]	0.9121	1.92	2.8273(13)	173
N4 - H4B...N2	0.9458	2.47	2.8487(14)	104
N5 - H5A...O2[d]	0.9544	1.89	2.8317(12)	167
N5 - H5B...O3[c]	0.9284	1.94	2.8572(12)	170
C4 - H4...N3	0.9500	2.49	3.0147(16)	115

Note. Equivalent positions: (a) x, 1/2-y, 1/2+z; (b) x, -1/2-y, -1/2+z; (c) x, 1/2-y, -1/2+z; (d) 1-x, -1/2+y, 1/2-z

Table S6. Selected bond lengths [Å] and angles [°] for **phbigua$_2$SO$_4$**.

Bond/Angle	Value (Å/°)	Angle	Value (°)
N1 - C1	1.347(3)	N3 - C1 - N1	119.0(2)
N1 - C3	1.420(3)	N2 - C1 - N1	115.67(19)
N2 - C1	1.338(3)	N4 - C2 - N5	119.2(2)
N3 - C1	1.316(3)	N4 - C2 - N3	122.9(2)
N3 - C2	1.346(3)	N5 - C2 - N3	117.9(2)
N4 - C2	1.335(4)	C3 - C4 - C5	119.7(3)
N4 - H4B	0.9592	C4 - C3 - N1	118.0(3)
N5 - C2	1.322(3)	C6 - C5 - C4	119.6(4)
C3 - C4	1.370(4)	C6 - C7 - C8	121.0(4)
C3 - C8	1.384(4)	C7 - C6 - C5	120.2(4)
C4 - C5	1.414(5)	C7 - C8 - C3	119.6(4)
C5 - C6	1.369(7)	C8 - C3 - C4	119.9(3)
C6 - C7	1.358(8)	C8 - C3 - N1	122.0(3)
C7 - C8	1.384(5)	O1^d - S1 - O1	113.3(2)
S1 - O1	1.460(2)	O1^d - S1 - O2^d	109.42(11)
S1 - O2	1.4704(19)	O1 - S1 - O2^d	109.12(17)
C1 - N1 - C3	126.56(19)	O1^d - S1 - O2	109.13(17)
C1 - N3 - C2	121.8(2)	O1 - S1 - O2	108.41(11)
N3 - C1 - N2	125.1(2)	O2^d - S1 - O2	108.35(17)

Hydrogen bonds

D-H...A	d(D-H)	d(H...A)	d(D...A)	<(DHA)
N1 - H1...O2	0.9802	1.84	2.813(2)	170
N2 - H2A…N4	0.9769	2.53	2.926(4)	104
N2 - H2B...O1	0.9181	1.93	2.852(2)	176
N4 - H4A...O1^a	0.8824	2.29	3.066(4)	147
N5 - H5A...O1^b	0.9344	2.02	2.849(4)	146
N5 - H5B...O2^c	0.9737	1.89	2.857(2)	171
C8 - H8…N3	0.9300	2.45	2.882(4)	108

Note. Equivalent positions: (a) 3/2-x, 1/2+y, -z; (b) 3/2-x, -1/2+y, -z; (c) 1/2+x, 1/2+y, z; (d) 1-x, y, -z

Table S7. Selected bond lengths [Å] and angles [°] for **phbigua$_2$HPO$_4$1.5H$_2$O**.

Bond	Value (Å)	Bond	Value (Å)	Bond	Value (Å)	Bond/Angle	Value (Å/°)	Angle	Value (°)	Angle	Value (°)	Angle	Value (°)
N11 - C11	1.355(3)	N41 - C41	1.348(2)	N71 - C71	1.342(3)	P41 - O43	1.524(2)	N32 - C31 - N31	115.9(2)	C54 - C53 - N51	122.8(2)	C81 - N83 - C82	122.1(2)
N11 - C13	1.420(3)	N41 - C43	1.418(3)	N71 - C73	1.422(3)	P41 - O41	1.525(2)	N33 - C31 - N31	118.6(2)	C55 - C54 - C53	120.1(2)	N83 - C81 - N82	125.4(2)
N12 - C11	1.324(3)	N42 - C41	1.335(3)	N72 - C71	1.342(3)	P41 - O44	1.583(2)	N34 - C32 - N33	117.7(2)	C54 - C55 - C56	120.7(2)	N83 - C81 - N81	119.1(2)
N13 - C11	1.334(3)	N43 - C41	1.333(3)	N73 - C71	1.336(3)			N34 - C32 - N35	117.5(2)	C57 - C56 - C55	119.2(2)	N82 - C81 - N81	115.4(2)
N13 - C12	1.342(3)	N43 - C42	1.342(3)	N73 - C72	1.343(3)	C11 - N11 - C13	127.7(2)	N33 - C32 - N35	124.8(2)	C56 - C57 - C58	120.7(2)	N84 - C82 - N85	117.3(2)
N14 - C12	1.324(3)	N44 - C42	1.313(3)	N74 - C72	1.331(3)	C11 - N13 - C12	122.6(2)	C34 - C33 - C38	118.7(2)	C53 - C58 - C57	119.8(2)	N84 - C82 - N83	117.7(2)
N15 - C12	1.344(3)	N45 - C42	1.343(3)	N75 - C72	1.336(3)	N12 - C11 - N13	125.8(2)	C34 - C33 - N31	117.8(2)	C61 - N61 - C63	126.5(2)	N85 - C82 - N83	124.9(2)
C13 - C18	1.391(3)	C43 - C44	1.394(3)	C73 - C74	1.390(3)	N12 - C11 - N11	116.1(2)	C38 - C33 - N31	123.4(2)	C61 - N63 - C62	123.6(2)	C88 - C83 - C84	119.6(2)
C13 - C14	1.394(3)	C43 - C48	1.399(3)	C73 - C78	1.391(3)	N13 - C11 - N11	118.0(2)	C35 - C34 - C33	120.6(2)	N62 - C61 - N63	124.3(2)	C88 - C83 - N81	124.2(2)
C14 - C15	1.384(3)	C44 - C45	1.391(3)	C74 - C75	1.389(3)	N14 - C12 - N13	117.3(2)	C36 - C35 - C34	120.7(2)	N62 - C61 - N61	116.3(2)	C84 - C83 - N81	116.1(2)
C15 - C16	1.388(3)	C45 - C46	1.380(3)	C75 - C76	1.387(3)	N14 - C12 - N15	117.8(2)	C35 - C36 - C37	118.7(2)	N63 - C61 - N61	119.3(2)	C85 - C84 - C83	120.0(2)
C16 - C17	1.387(3)	C46 - C47	1.383(3)	C76 - C77	1.381(3)	N13 - C12 - N15	124.8(2)	C38 - C37 - C36	121.2(2)	N64 - C62 - N63	117.6(2)	C86 - C85 - C84	120.4(2)
C17 - C18	1.388(3)	C47 - C48	1.381(3)	C77 - C78	1.385(3)	C18 - C13 - C14	119.6(2)	C37 - C38 - C33	120.0(2)	N64 - C62 - N65	117.7(2)	C87 - C86 - C85	119.3(2)
N21 - C21	1.345(3)	N51 - C51	1.341(3)	N81 - C81	1.361(3)	C18 - C13 - N11	116.7(2)	C41 - N41 C43	129.6(2)	N63 - C62 - N65	124.6(2)	C86 - C87 - C88	121.3(2)
N21 - C23	1.419(3)	N51 - C53	1.418(3)	N81 - C83	1.415(3)	C14 - C13 - N11	123.6(2)	C41 - N43 - C42	123.2(2)	C64 - C63 - C68	119.4(2)	C87 - C88 - C83	119.3(2)
N22 - C21	1.335(3)	N52 - C51	1.338(3)	N82 - C81	1.331(3)	C15 - C14 - C13	119.5(2)	N43 - C41 - N42	124.2(2)	C64 - C63 - N61	123.6(2)	O13 - P11 - O11	113.15(8)
N23 - C21	1.338(3)	N53 - C52	1.337(3)	N83 - C81	1.329(3)	C14 - C15 - C16	121.0(2)	N43 - C41 - N41	119.6(2)	C68 - C63 - N61	116.8(2)	O13 - P11 - O12	113.19(8)
N23 - C22	1.341(3)	N53 - C51	1.340(3)	N83 - C82	1.350(3)	C17 - C16 - C15	119.3(2)	N42 - C41 - N41	116.0(2)	C65 - C64 - C63	120.0(2)	O11 - P11 - O12	111.05(8)
N24 - C22	1.325(3)	N54 - C52	1.322(3)	N84 - C82	1.327(3)	C16 - C17 - C18	120.2(2)	N44 - C42 - N43	118.7(2)	C64 - C65 - C66	120.9(2)	O13 - P11 - O14	103.50(8)
N25 - C22	1.336(3)	N55 - C52	1.339(3)	N85 - C82	1.336(3)	C17 - C18 - C13	120.3(2)	N44 - C42 - N45	116.7(2)	C67 - C66 - C65	118.9(2)	O11 - P11 - O14	106.97(8)
C23 - C28	1.386(3)	C53 - C58	1.388(3)	C83 - C88	1.393(3)	C21 - N21 - C23	128.3(2)	N43 - C42 - N45	124.3(2)	C66 - C67 - C68	121.0(2)	O12 - P11 - O14	108.41(8)
C23 - C24	1.392(3)	C53 - C54	1.391(3)	C83 - C84	1.398(3)	C21 - N23 - C22	124.0(2)	C44 - C43 - C48	119.0(2)	C67 - C68 - C63	119.8(2)	O22 - P21 - O23	113.07(8)
C24 - C25	1.383(3)	C54 - C55	1.382(3)	C84 - C85	1.390(3)	N22 - C21 - N23	126.1(2)	C44 - C43 - N41	125.1(2)	C71 - N71 - C73	127.3(2)	O22 - P21 - O21	113.28(8)
C25 - C26	1.385(3)	C55 - C56	1.385(4)	C85 - C86	1.384(3)	N22 - C21 - N21	115.5(2)	C48 - C43 - N41	115.9(2)	C71 - N73 - C72	123.6(2)	O23 - P21 - O21	111.18(8)
C26 - C27	1.380(3)	C56 - C57	1.383(3)	C86 - C87	1.383(3)	N23 - C21 - N21	118.3(2)	C45 - C44 - C43	119.6(2)	N73 - C71 - N72	125.1(2)	O22 - P21 - O24	103.61(8)
C27 - C28	1.387(3)	C57 - C58	1.389(3)	C87 - C88	1.390(3)	N24 - C22 - N25	117.9(2)	C46 - C45 - C44	121.1(2)	N73 - C71 - N71	118.6(2)	O23 - P21 - O24	106.73(8)
N31 - C31	1.342(3)	N61 - C61	1.351(3)	P11 - O13	1.513(1)	N24 - C22 - N23	116.4(2)	C45 - C46 - C47	119.2(2)	N72 - C71 - N71	116.3(2)	O21 - P21 - O24	108.37(8)
N31 - C33	1.417(3)	N61 - C63	1.424(3)	P11 - O11	1.525(2)	N25 - C22 - N23	125.7(2)	C48 - C47 - C46	120.6(2)	N74 - C72 - N75	118.0(2)	O31 - P31 - O33	110.59(8)
N32 - C31	1.336(3)	N62 - C61	1.331(3)	P11 - O12	1.526(2)	C28 - C23 - C24	119.0(2)	C47 - C48 - C43	120.3(2)	N74 - C72 - N73	115.9(2)	O31 - P31 - O32	111.16(8)
N33 - C32	1.332(3)	N63 - C61	1.333(3)	P11 - O14	1.608(1)	C28 - C23 - N21	124.0(2)	C51 - N51 - C53	126.9(2)	N75 - C72 - N73	126.1(2)	O33 - P31 - O32	112.19(8)
N33 - C31	1.339(3)	N63 - C62	1.342(3)	P21 - O22	1.517(2)	C24 - C23 - N21	116.9(2)	C52 - N53 - C51	123.8(2)	C74 - C73 - C78	119.5(2)	O31 - P31 - O34	106.84(8)
N34 - C32	1.324(3)	N64 - C62	1.321(3)	P21 - O23	1.525(2)	C25 - C24 - C23	120.5(2)	N52 - C51 - N53	124.9(2)	C74 - C73 - N71	117.3(2)	O33 - P31 - O34	107.99(8)
N35 - C32	1.339(3)	N65 - C62	1.343(3)	P21 - O21	1.527(2)	C24 - C25 - C26	120.5(2)	N52 - C51 - N51	116.4(2)	C78 - C73 - N71	123.1(2)	O32 - P31 - O34	107.82(8)
C33 - C34	1.387(3)	C63 - C64	1.389(3)	P21 - O24	1.606(1)	C27 - C26 - C25	118.9(2)	N53 - C51 - N51	118.6(2)	C75 - C74 - C73	119.9(2)	O42 - P41 - O43	111.60(9)
C33 - C38	1.394(3)	C63 - C68	1.392(3)	P31 - O31	1.519(1)	C26 - C27 - C28	121.2(2)	N54 - C52 - N53	117.8(2)	C76 - C75 - C74	120.8(2)	O42 - P41 - O41	110.66(9)
C34 - C35	1.386(3)	C64 - C65	1.384(3)	P31 - O33	1.525(1)	C23 - C28 - C27	119.9(2)	N54 - C52 - N55	116.8(2)	C77 - C76 - C75	118.9(2)	O43 - P41 - O41	110.61(9)
C35 - C36	1.379(3)	C65 - C66	1.385(3)	P31 - O32	1.528(1)	C31 - N31 - C33	127.7(2)	N53 - C52 - N55	125.4(2)	C76 - C77 - C78	121.1(2)	O42 - P41 - O44	108.29(8)
C36 - C37	1.386(4)	C66 - C67	1.381(3)	P31 - O34	1.597(1)	C32 - N33 - C31	124.7(2)	C58 - C53 - C54	119.5(2)	C77 - C78 - C73	119.8(2)	O43 - P41 - O44	109.27(8)
C37 - C38	1.380(3)	C67 - C68	1.386(3)	P41 - O42	1.520(2)	N32 - C31 - N33	125.4(2)	C58 - C53 - N51	117.6(2)	C81 - N81 - C83	129.3(2)	O41 - P41 - O44	106.24(8)

Table S7. Selected bond lengths [Å] and angles [°] for **phbigua$_2$HPO$_4$1.5H$_2$O** - continued.

Hydrogen bonds					Hydrogen bonds									
**D-H...A**	**d(D-H)**	**d(H...A)**	**d(D...A)**	**<(DHA)**	**D-H...A**	**d(D-H)**	**d(H...A)**	**d(D...A)**	**<(DHA)**	**D-H...A**	**d(D-H)**	**d(H...A)**	**d(D...A)**	**<(DHA)**
N11 - H11...O14	0.86	2.41	3.185(2)	150	N45 - H45B...N42	0.86	2.54	2.956(3)	111	N84 - H84A...O13[e]	0.86	2.59	3.284(2)	139
N12 - H12A...N63[a]	0.86	2.25	3.033(2)	152	N51 - H51...O31	0.86	1.88	2.738(2)	172	N84 - H84B...O4W	0.86	2.19	2.891(2)	138
N12 - H12B...O11	0.86	1.92	2.759(2)	164	N52 - H52A...N33[f]	0.86	2.25	3.037(2)	153	N85 - H85A...O13[e]	0.86	2.01	2.843(2)	161
N14 - H14A...O23[a]	0.86	2.20	2.982(2)	151	N52 - H52B...O33	0.86	2.28	3.067(2)	153	N85 - H85B...N82	0.86	2.44	2.886(3)	113
N14 - H14B...O42[a]	0.86	1.93	2.759(2)	160	N52 - H52B...O31	0.86	2.57	3.242(2)	136	O14 - H14...O12[j]	1.00	1.65	2.635(2)	171
N15 - H15A...O41[a]	0.86	2.12	2.976(2)	178	N54 - H54A...O42[f]	0.86	2.06	2.877(2)	158	O24 - H24...O21[c]	1.00	1.66	2.635(2)	164
N15 - H15B...N12	0.86	2.46	2.920(3)	114	N54 - H54B...O21[f]	0.86	2.15	2.965(2)	157	O34 - H34...O32[k]	0.90	1.73	2.624(2)	173
N21 - H21...O13	0.86	1.96	2.777(2)	158	N55 - H55A...O21[f]	0.86	2.37	3.127(2)	147	O44 - H44...O43[l]	0.95	1.60	2.537(2)	168
N22 - H22A...N73[b]	0.86	2.33	3.141(3)	157	N55 - H55B...N52	0.86	2.43	2.921(3)	117	O1W - H1W1...O41	0.98	1.81	2.787(2)	172
N22 - H22B...O13	0.86	2.19	2.939(2)	145	N61 - H61...O43	0.86	2.00	2.830(2)	162	O2W - H2W1...O42	0.90	2.00	2.883(2)	167
N24 - H24A...O44[b]	0.86	2.21	3.003(2)	154	N62 - H62A...N43[g]	0.86	2.43	3.220(3)	152	O2W - H2W2...O23	0.93	1.87	2.799(2)	174
N24 - H24A...O2W[c]	0.86	2.57	3.090(2)	120	N62 - H62B...O1W	0.86	2.03	2.808(3)	150	O3W - H3W1...O23	0.97	1.94	2.895(2)	170
N24 - H24B...O3W[c]	0.86	2.22	2.859(3)	131	N64 - H64A...O11[e]	0.86	2.27	3.068(2)	154	O3W - H3W2...O21[c]	1.02	1.76	2.763(2)	170
N25 - H25A...O44[b]	0.86	2.31	3.080(2)	149	N64 - H64B...O33[g]	0.86	1.93	2.776(2)	167	O4W - H4W1...O5W[k]	0.95	1.80	2.744(2)	173
N25 - H25B...N22	0.86	2.42	2.942(3)	120	N65 - H65A...O31[g]	0.86	2.15	3.007(2)	174	O4W - H4W2...O32	0.85	2.06	2.904(2)	170
N31 - H31...O22	0.86	1.96	2.782(2)	160	N65 - H65B...N62	0.86	2.46	2.930(3)	115	O5W - H5W1...O33	0.87	1.92	2.788(2)	177
N32 - H32A...N23	0.86	2.33	3.153(3)	160	N71 - H71...O41	0.86	1.87	2.721(2)	173	O5W - H5W2...O11[f]	0.87	1.90	2.770(2)	177
N32 - H32B...O22	0.86	2.23	2.971(2)	144	N72 - H72A...N53[d]	0.86	2.34	3.111(2)	149	O6W - H6W1...O12[j]	0.92	1.84	2.751(2)	174
N34 - H34A...O33[d]	0.86	2.10	2.921(2)	159	N72 - H72A...N75	0.86	2.46	2.891(3)	111	O6W - H6W2...O11	0.92	2.00	2.892(2)	164
N34 - H34B...O12	0.86	2.10	2.931(2)	162	N72 - H72B...O42	0.86	2.14	2.949(2)	157	O1W - H1W2...O2W[l]	0.99	1.89	2.787(3)	149
N35 - H35A...O12	0.86	2.52	3.243(2)	143	N74 - H74A...O34[d]	0.86	2.41	3.154(2)	145	C18 - H18…O4W[a]	0.93	2.56	3.161(3)	123
N35 - H35B...N32	0.86	2.46	2.966(3)	118	N74 - H74A...O5W[h]	0.86	2.43	2.957(2)	120	C14-H14C...N13	0.93	2.46	2.909(3)	109
N41 - H41...O32	0.86	1.98	2.836(2)	175	N74 - H74B...O6W[i]	0.86	2.24	2.888(3)	132	C28-H28...N23	0.93	2.39	2.892(3)	114
N42 - H42A...N83	0.86	2.26	3.057(2)	155	N74 - H74A...O34[d]	0.86	2.41	3.154(2)	145	C38-H38...N33	0.93	2.43	2.908(3)	112
N42 - H42B...O31	0.86	2.39	2.994(2)	128	N75 - H75B...N72	0.86	2.37	2.891(3)	119	C44-H44C...N43	0.93	2.32	2.883(3)	119
N42 - H42B...O4W	0.86	2.40	3.092(2)	138	N81 - H81...O24	0.86	2.29	3.091(2)	154	C54-H54...N53	0.93	2.47	2.905(3)	109
N44 - H44A...O1W[b]	0.86	1.87	2.686(3)	158	N82 - H82A...N13[e]	0.86	2.29	3.066(2)	150	C64-H64...N63	0.93	2.41	2.880(3)	111
N44 - H44B...O22	0.86	2.21	2.992(3)	151	N82 - H82A...N85	0.86	2.46	2.886(3)	111	C78-H78...N73	0.93	2.44	2.891(3)	110
N44 - H44B...O24	0.86	2.29	2.992(2)	139	N82 - H82B...O23	0.86	1.96	2.799(2)	165	C88-H88...N83	0.93	2.42	2.928(3)	114
N45 - H45A...O22	0.86	2.30	3.062(2)	148	N84 - H84A...O14[e]	0.86	2.21	2.971(2)	147					

Note. Equivalent positions: (a) -1+x,-1+y, z; (b) -1+x, y, z; (c) 1-x, 1-y, 2-z; (d) x, -1+y, z; (e) 1+x, 1+y, z; (f) x, 1+y, z; (g) 1+x, y, z; (h) 1-x, 1-y, 1-z; (i) 1-x, -y, 1-z; (j) -x, -y, 1-z; (k) 1-x, 2-y, 1-z; (l) 2-x, 1-y, 2-z

Table S8. Selected bond lengths [Å] and angles [°] for **phbigua$_2$HPO$_3$**.

Bond	Value (Å)	Angle	Value (°)
P1 - O1	1.5087(13)	O1 - P1 - O3	118.00(8)
P1 - O3	1.5114(14)	O1 - P1 - O2	110.73(7)
P1 - O2	1.5360(13)	O3 - P1 - O2	110.27(7)
P1 - H1	1.36(2)	O1 - P1 - H1	105.2(10)
C11 - N13	1.321(2)	O3 - P1 - H1	103.1(10)
C11 - N12	1.346(2)	O2 - P1 - H1	108.7(9)
C11 - N11	1.352(2)	N13 - C11 - N12	125.75(17)
N11 - C13	1.411(2)	N13 - C11 - N11	118.91(17)
C12 - N15	1.321(2)	N12 - C11 - N11	115.20(15)
C12 - N13	1.344(2)	C11 - N11 - C13	128.21(15)
C12 - N14	1.351(2)	N15 - C12 - N13	117.16(16)
C13 - C14	1.396(2)	N15 - C12 - N14	118.83(16)
C13 - C18	1.397(3)	N13 - C12 - N14	123.99(16)
C14 - C15	1.387(3)	C14 - C13 - C18	119.27(18)
N14 - H14A	0.8811	C14 - C13 - N11	116.54(17)
C15 - C16	1.383(3)	C18 - C13 - N11	123.99(16)
C16 - C17	1.389(3)	C11 - N13 - C12	122.71(16)
C17 - C18	1.391(3)	C15 - C14 - C13	120.54(18)
C21 - N23	1.319(2)	C16 - C15 - C14	120.52(19)
C21 - N22	1.345(2)	C15 - C16 - C17	119.0(2)
C21 - N21	1.355(2)	C16 - C17 - C18	121.3(2)
N21 - C23	1.414(2)	C17 - C18 - C13	119.34(19)
C22 - N25	1.321(2)	N23 - C21 - N22	125.64(17)
C22 - N24	1.340(2)	N23 - C21 - N21	119.05(16)
C22 - N23	1.351(2)	N22 - C21 - N21	115.23(16)
C23 - C24	1.389(2)	C21 - N21 - C23	124.56(15)
C23 - C28	1.396(2)	N25 - C22 - N24	119.11(16)
C24 - C25	1.392(3)	N25 - C22 - N23	117.47(16)
N24 - H24A	0.8514	N24 - C22 - N23	123.37(16)
C25 - C26	1.376(3)	C24 - C23 - C28	119.55(16)
C26 - C27	1.388(3)	C24 - C23 - N21	117.94(16)
C27 - C28	1.388(3)	C28 - C23 - N21	122.39(16)
		C21 - N23 - C22	122.34(16)
		C23 - C24 - C25	120.47(18)

Hydrogen bonds				
**D-H...A**	**d(D-H)**	**d(H...A)**	**d(D...A)**	**<(DHA)**
N11 - H11...O2[a]	0.89	2.01	2.8668(19)	162
N12 - H12A...O1[a]	0.87	1.95	2.821(2)	179
N14 - H14B...O3[b]	0.94	2.22	3.156(2)	169
N15 - H15A...O3	0.86	1.93	2.7750(19)	166
N15 - H15B...O2[b]	0.90	1.94	2.8228(18)	167
N21 - H21...O3[c]	0.86	1.98	2.839(2)	172
N22 - H22A...O2[c]	0.89	2.13	2.960(2)	155
N22 - H22B...N24	0.92	2.50	2.947(2)	110
N24 - H24B...O1[b]	0.84	2.26	3.098(2)	174
N25 - H25A...O1	0.88	1.92	2.783(2)	166
N25 - H25B...O2[b]	0.93	1.93	2.843(2)	169
C18-H18...N13	0.95	2.52	2.967(3)	109
C24-H24...N15[c]	0.95	2.54	3.292(2)	136
C27-H27...N15[d]	0.95	2.61	3.440(2)	147

Note. Equivalent positions: (a) 1+x, y, 1+z; (b) 1+x, y, z; (c) 1-x, -1/2+y, 1-z; (d) -1+x, y, -1+z

Table S9. Selected bond lengths [Å] and angles [°] for **phbigua$_2$HPO$_3$2H$_2$O**.

Bond/Angle	Value (Å/°)	Angle	Value (°)
P1 - O3	1.519(1)	O2 - P1 - H1	105.4(6)
P1 - O2	1.519(1)	O1 - P1 - H1	104.9(6)
P1 - O1	1.519(1)	C11 - N11 - C13	125.8(1)
P1 - H1	1.32(1)	C11 - N13 - C12	124.0(1)
N11 - C11	1.355(2)	N13 - C11 - N12	125.5(1)
N11 - C13	1.418(2)	N13 - C11 - N11	118.2(1)
N12 - C11	1.333(2)	N12 - C11 - N11	116.2(1)
N13 - C11	1.329(2)	N15 - C12 - N13	117.4(1)
N13 - C12	1.335(2)	N15 - C12 - N14	118.5(1)
N14 - C12	1.350(2)	N13 - C12 - N14	124.1(1)
N15 - C12	1.322(2)	C14 - C13 - C18	119.7(1)
C13 - C14	1.387(2)	C14 - C13 - N11	117.0(1)
C13 - C18	1.392(2)	C18 - C13 - N11	123.4(1)
C14 - C15	1.377(2)	C15 - C14 - C13	120.3(1)
C15 - C16	1.383(2)	C14 - C15 - C16	120.5(1)
C16 - C17	1.384(2)	C15 - C16 - C17	119.1(1)
C17 - C18	1.382(2)	C18 - C17 - C16	121.1(1)
N21 - C21	1.347(2)	C17 - C18 - C13	119.3(1)
N21 - C23	1.422(2)	C21 - N21 - C23	127.2(1)
N22 - C21	1.337(2)	C21 - N23 - C22	123.1(1)
N23 - C21	1.332(2)	N23 - C21 - N22	124.9(1)
N23 - C22	1.342(2)	N23 - C21 - N21	118.9(1)
N24 - C22	1.339(2)	N22 - C21 - N21	116.2(1)
N25 - C22	1.328(2)	N25 - C22 - N24	117.7(1)
C23 - C28	1.386(2)	N25 - C22 - N23	117.3(1)
C23 - C24	1.389(2)	N24 - C22 - N23	124.9(1)
C24 - C25	1.388(2)	C28 - C23 - C24	119.4(1)
C25 - C26	1.377(2)	C28 - C23 - N21	122.8(1)
C26 - C27	1.383(2)	C24 - C23 - N21	117.6(1)
C27 - C28	1.381(2)	C25 - C24 - C23	119.9(1)
O3 - P1 - O2	111.75(6)	C26 - C25 - C24	120.6(1)
O3 - P1 - O1	112.91(6)	C25 - C26 - C27	119.3(1)
O2 - P1 - O1	113.87(6)	C28 - C27 - C26	120.7(2)
O3 - P1 - H1	107.2(6)	C27 - C28 - C23	120.0(1)

Hydrogen bonds				
**D-H...A**	**d(D-H)**	**d(H...A)**	**d(D...A)**	**<(DHA)**
N11 - H11...O2[a]	0.92	2.03	2.912(2)	160
N12 - H12A...O3[a]	0.86	1.93	2.784(2)	175
N12 - H12B...N13[a]	0.87	2.58	3.169(2)	125
N14 - H14B...O2W[b]	0.87	2.19	3.016(2)	160
N14 - H14A...N12	0.91	2.51	2.911(2)	107
N15 - H15A...O3	0.92	1.95	2.864(2)	175
N15 - H15B...O1W[b]	0.86	1.99	2.832(2)	169
N21 - H21...O2W	0.91	1.99	2.887(2)	166
N22 - H22A...O1[c]	0.92	2.03	2.906(2)	159
N22 - H22B...N23[d]	0.91	2.39	3.172(2)	144
N24 - H24A...N22	0.90	2.52	2.897(2)	106
N24 - H24B...O2[e]	0.94	1.91	2.857(2)	179
N25 - H25A...O1W[e]	0.91	1.99	2.887(2)	169
N25 - H25B...O1[f]	0.92	2.09	2.960(2)	158
O1W - H1A...O2	0.94	1.80	2.685(1)	155
O1W - H1B...O1[c]	0.89	1.95	2.812(1)	162
O2W - H2A...O1[c]	0.91	1.84	2.720(1)	163
O2W - H2B...O3	0.92	1.84	2.743(1)	165
C18 - H18…N13	0.93	2.56	2.911(2)	103
C28 - H28…N23	0.93	2.46	2.901(2)	109

Note. Equivalent positions: (a) 1/2-x, ½+y, 3/2-z; (b) x, 1+y, z; (c) 1-x, -y, 1-z; (d) 1/2-x, -1/2-y, -1/2+z; (e) -1/2+x, -1/2-y, -1/2+z; (f) -1/2+x, 1/2-y, -1/2+z

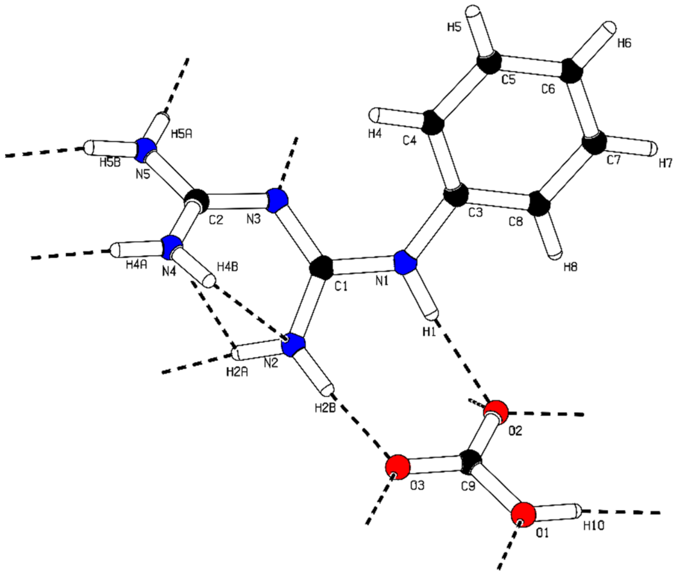

Figure S1. Atom numbering of **phbiguaNO₃**. Dashed lines indicate hydrogen bonds.

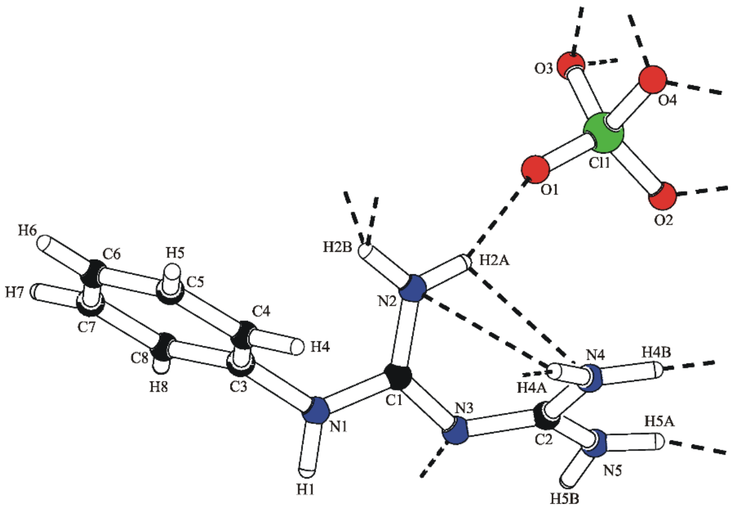

Figure S2. Atom numbering of **phbiguaClO₄**. Dashed lines indicate hydrogen bonds.

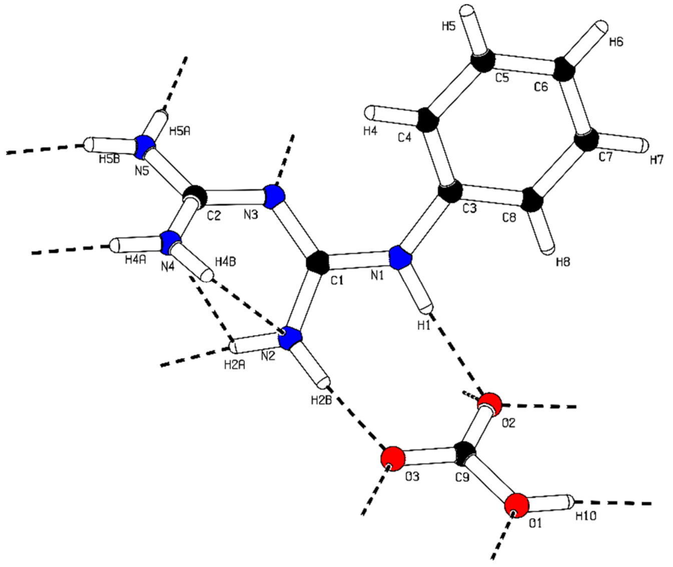

Figure S3. Atom numbering of **phbiguaHCO$_3$**. Dashed lines indicate hydrogen bonds.

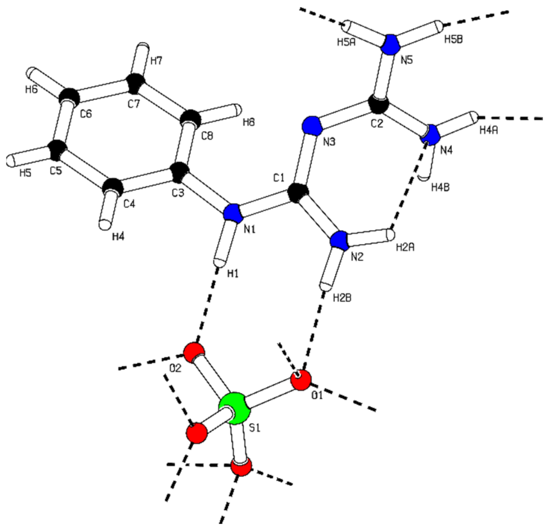

Figure S4. Atom numbering of **phbigua$_2$SO$_4$**. Dashed lines indicate hydrogen bonds.

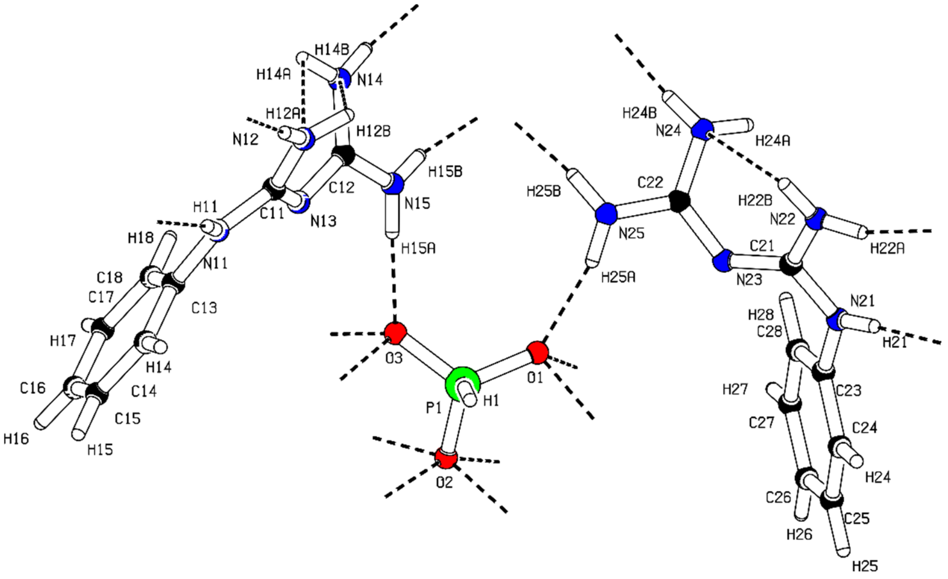

Figure S5. Atom numbering of **phbigua$_2$HPO$_3$**. Dashed lines indicate hydrogen bonds.

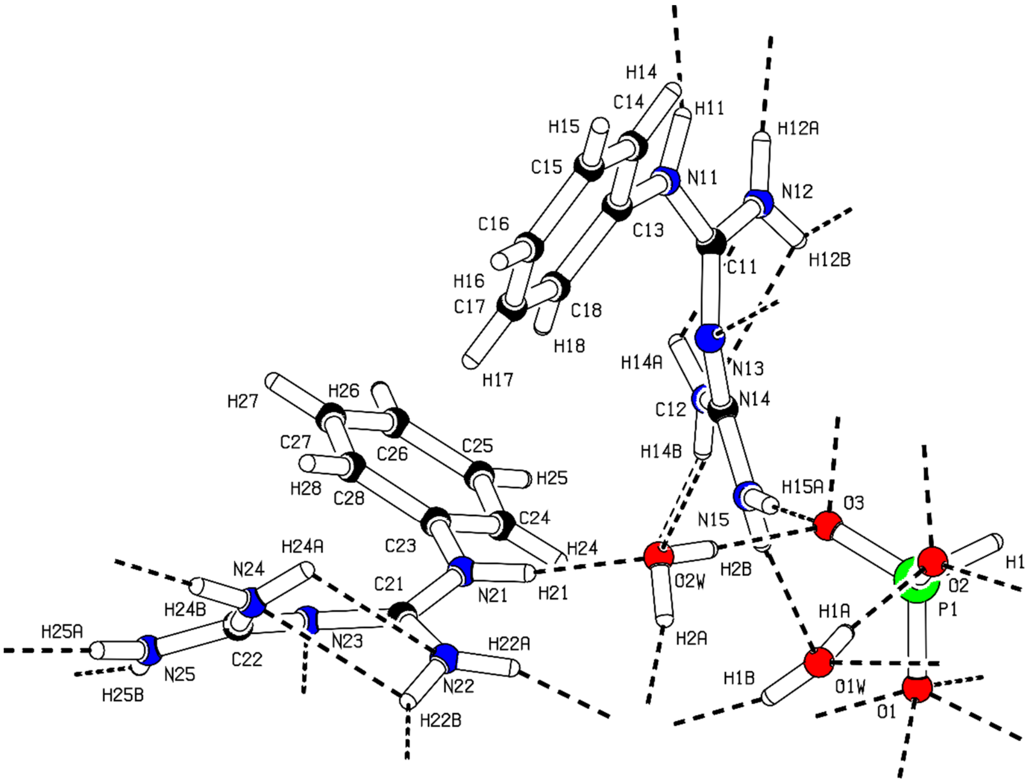

Figure S6. Atom numbering of **phbigua$_2$HPO$_3$2H$_2$O**. Dashed lines indicate hydrogen bonds.

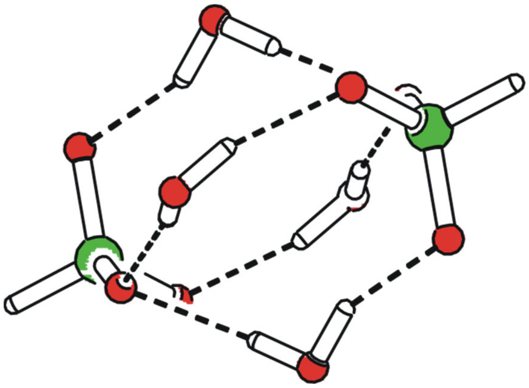

Figure S7. Detail of pairs of phosphite anions and water molecules inter-connected via hydrogen bonds in the crystal structure of **phbigua$_2$HPO$_3$2H$_2$O**.

Listing S1. List of recorded IR and Raman maxima (cm^{-1}) of **phbiguaNO$_3$**, **phbiguaClO$_4$**, **phbiguaHCO$_3$**, **phbigua$_2$SO$_4$**, **phbigua$_2$HPO$_4$1.5H$_2$O**, **phbigua$_2$HPO$_3$** and **phbigua$_2$HPO$_3$2H$_2$O**.

PhbiguaNO$_3$ FTIR: 3468 m; 3430 s; 3333 s; 3215 s; 2724 w; 2416 w; 1776 w; 1662 s; 1650 s; 1629 s; 1603 m; 1583 m; 1530 vs; 1496 s; 1451 s; 1397 m; 1371 s; 1308 m; 1295 m; 1259 m; 1190 m; 1157 w; 1081 m; 1059 m; 1052 m; 983 w; 929 w; 907 w; 846 w; 839 w; 821 m; 763 m; 748 m; 724 m; 716 m; 696 m; 638 m; 615 m; 601 m; 523 m; 501 m; 491 m; 405 m.
FT Raman: 3350 wb; 3305 wb; 3240 wb; 3225 wb; 3066 m; 3057 m; 1664 w; 1634 m; 1602 s; 1585 m; 1555 m; 1498 m; 1450 w; 1375 w; 1295 s; 1259 s; 1177 m; 1158 m; 1081 w; 1056 vs; 1027 m; 1004 s; 967 w; 928 m; 846 m; 762 w; 745 m; 724 m; 678 w; 634 w; 614 m; 526 w; 501 w; 492 w; 409 w; 399 w; 382 w; 335 w; 270 m; 227 m; 148 m; 132 s.

PhbiguaClO$_4$ FTIR: 3424 s; 3379 s; 3352 s; 3250 s; 3143 s; 3055 m; 2756 w; 2468 w; 2003 w; 1672 s; 1636 s; 1597 m; 1576 s; 1530 vs; 1493 s; 1452 m; 1415 w; 1380 m; 1238 m; 1122 sh; 1085 s; 1028 m; 943 w; 935 w; 923 w; 853 w; 783 w; 752 w; 726 m; 699 m; 673 w; 624 m; 600 wb; 502 m; 460 w. FT Raman: 3376 w; 3354 wb; 3249 wb; 3070 s; 3013 w; 2975 vw; 1677 vw; 1638 w; 1596 m; 1584 w; 1569 w; 1550 w; 1527 w; 1450 w; 1314 w; 1233 m; 1174 w; 1158 w; 1125 wb; 1070 wb; 1026 w; 1000 s; 944 s; 935 vs; 857 w; 753 w; 705 w; 665 w; 625 m; 615 sh; 533 w; 506 vw; 461 m; 414 w; 396 w; 318 w; 289 w; 235 m; 140 m.

PhbiguaHCO$_3$ FTIR: 3467 s; 3293 sb; 3219 s; 3083 sb; 3018 sb 2806 mb; 2573 wb; 2346 w; 1953 w; 1702 m; 1652 sb; 1625 m; 1601 m; 1579 m; 1520 sh; 1498 s; 1487 s; 1452 m; 1404 m; 1346 m; 1306 m; 1258 w; 1200 m; 1178 w; 1147 w; 1097 w; 1068 w; 1015 m; 941 m; 904 m; 859 m; 843 m; 753 s; 689 s; 673 s; 637 m; 545 m; 504 m; 442 m; 410 w. FT Raman: 3467 w; 3220 w; 3064 s; 3037 w; 2560 vw; 1697 w; 1655 w; 1602 vs; 1555 m; 1531 w; 1502 m; 1487 w; 1451 w; 1343 w; 1299 s; 1256 m; 1200 w; 1177 m; 1158 m; 1088 m; 1065 m; 1028 s; 1012 s; 998 vs; 939 m; 865 m; 749 m; 723 w; 682 w; 636 m; 614 m; 546 m; 506 m; 436 w; 398 w; 326 m; 299 m; 248 m; 207 w; 183 w.

Phbigua$_2$SO$_4$ FTIR: 3465 m; 3349 m; 3290 mb; 3125 sb; 2990 mb; 2730 mb; 1720 sh; 1670 sh; 1637 s; 1602 m; 1582 m; 1535 sb; 1497 s; 1451 m; 1399 m; 1309 w; 1293 w; 1261 w; 1195 w; 1176 w; 1157 w; 1101 vs; 1077 sh; 1057 m; 975 w; 949 w; 907 w; 839 w; 766 m; 750 m; 724 m; 694 s; 674 sh; 622 m; 610 m; 535 m; 505 m; 492 sh. FT Raman: 3450 wb; 3300 wb; 3170 wb; 3092 w; 3068 m; 3060 sh; 3035 w; 2990 wb; 1675 w; 1640 w; 1629 w; 1602 s; 1586 m; 1557 m; 1497 w; 1450 w; 1292 s; 1261 s; 1176 w; 1157 m; 1078 w; 1058 w; 1025 m; 1002 vs; 980 vs; 926 m; 842 m; 767 w; 749 m; 673 w; 613 m; 532 w; 504 w; 469 w; 432 w; 409 w; 379 w; 340 w; 266 m; 228 w; 185 m; 152 m; 133 m.

Phbigua$_2$HPO$_4$1.5H$_2$O FTIR: 3447 m; 3335 mb; 3305 sh; 3186 mb; 3060 sh; 2890 mb; 2430 wb; 1644 s; 1604 s; 1585 s; 1575 s; 1555 s; 1535 vs; 1497 s; 1450 m; 1400 mb; 1296 w; 1261 m; 1200 wb; 1179 w; 1065 m; 1049 m; 989 m; 926 w; 908 w; 860 m; 838 m; 761 s; 737 m; 717 m; 695 m; 632 m; 594 w; 543 m; 521 m; 500 m; 485 m; 413 m. FT Raman: 3443 w; 3200 wb; 3074 m; 3061 m; 1655 w; 1602 s; 1554 m; 1498 m;

1447 w; 1327 w; 1296 s; 1262 s; 1178 m; 1163 w; 1154 m; 1081 w; 1057 w; 1029 m; 999 vs; 925 m; 915 m; 906 m; 855 m; 846 m; 754 m; 727 w; 675 w; 639 w; 616 m; 523 w; 502 m; 490 m; 411 m; 376 m; 319 w; 261 m; 238 w; 223 m; 149 s; 126 s.

Phbigua$_2$HPO$_3$ FTIR: 3469 m; 3445 m; 3300 mb; 3010 mb; 2284 m; 1654 sh; 1637 s; 1630 sh; 1602 s; 1583 s; 1534 vs; 1497 s; 1449 s; 1407 sh; 1396 m; 1294 w; 1263 wb; 1189 wb; 1125 m; 1090 m; 1076 sh; 1034 m; 1011 w; 989 w; 978 m; 922 w; 909 w; 863 sh; 812 mb; 769 m; 744 m; 716 m; 696 m; 628 m; 567 m; 544 m; 504 m; 453 m; 420 sh; 407 w. FT Raman: 3076 m; 3060 m; 2282 m; 1655 sh; 1628 sh; 1602 s; 1556 m; 1498 m; 1448 w; 1294 s; 1262 s; 1176 m; 1158 m; 1081 w; 1058 w; 1028 m; 1002 vs; 977 m; 923 m; 847 m; 747 m; 714 w; 678 w; 615 m; 545 w; 503 w; 491 w; 410 w; 380 w; 360 w; 339 w; 266 m; 238 m; 168 sh; 154 s.

Phbigua$_2$HPO$_3$2H$_2$O FTIR: 3442 m; 3310 m; 3097 sb; 2739 sh; 2347 m; 2287 w; 2040 w; 1943 w; 1869 w; 1684 sh; 1659 sb; 1648 m; 1637 m; 1630 m; 1619 sh; 1604 m; 1583 m; 1559 m; 1541 s; 1535 s; 1517 sb; 1497 s; 1483 sh; 1449 s; 1400 sb; 1311 w; 1296 m; 1257 m; 1190 mb; 1177 m; 1162 w; 1090 sb; 1072 s; 1052 s; 1039 s; 1028 m; 978 m; 924 w; 907 m; 833 m; 815 mb; 763 s; 738 m; 734 m; 722 m; 695 s; 680 sh; 632 m; 615 m; 560 m; 533 m; 495 mb; 467 m; 455 m; 426 m; 420 m. FT Raman: 3439 w; 3200 mb; 3080 m; 3067 m; 3060 m; 3051 sh; 2347 m; 1656 w; 1635 vw; 1602 vs; 1545 m; 1497 m; 1449 w; 1295 s; 1256 s; 1177 m; 1156 m; 1078 w; 1029 m; 1000 vs; 978 m; 923 m; 906 w; 841 m; 767 w; 744 w; 677 w; 631 w; 615 m; 532 w; 502 m; 431 w; 412 m; 379 w; 353 w; 274 m; 244 m; 190 m; 160 m; 138 s.

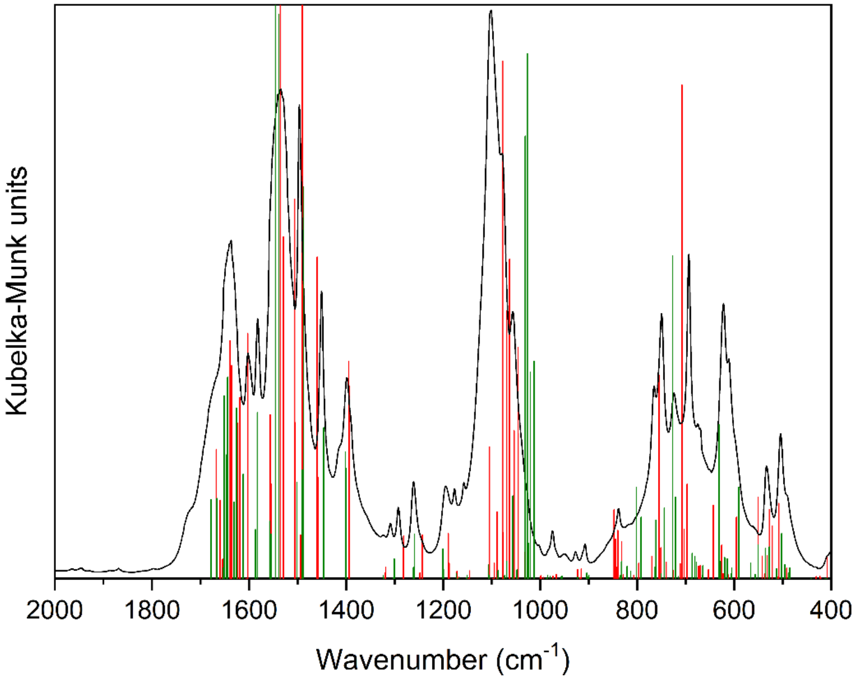

Figure S8. The comparison of calculated (CRYSTAL17 programme) vibrational modes using CAM-B3LYP (red lines) and B3LYP (green lines) functionals with recorded DRIFTS spectrum of **phbigua$_2$SO$_4$**.

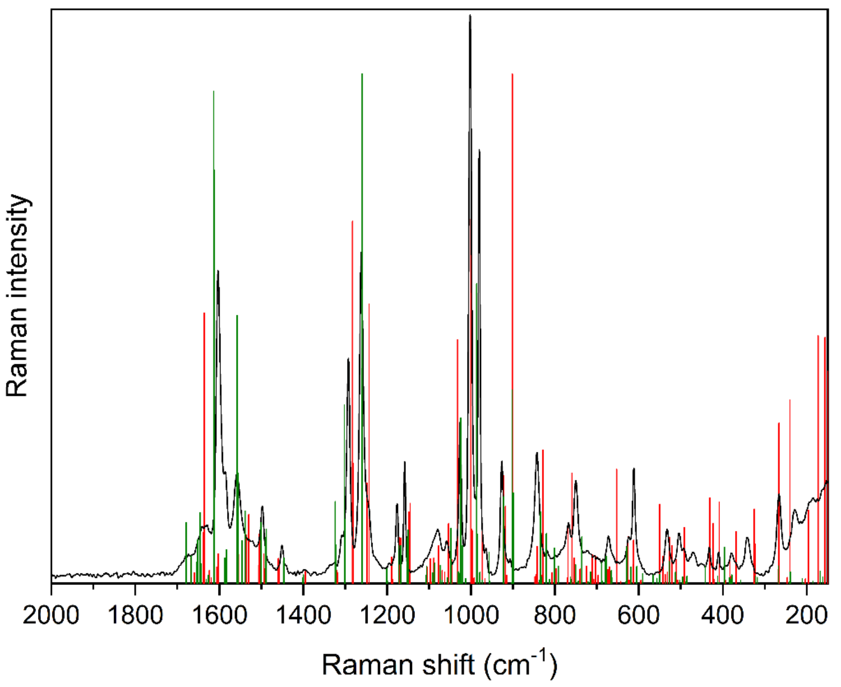

Figure S9. The comparison of calculated (CRYSTAL17 programme) vibrational modes using CAM-B3LYP (red lines) and B3LYP (green lines) functionals with recorded Raman spectrum of **phbigua$_2$SO$_4$**.

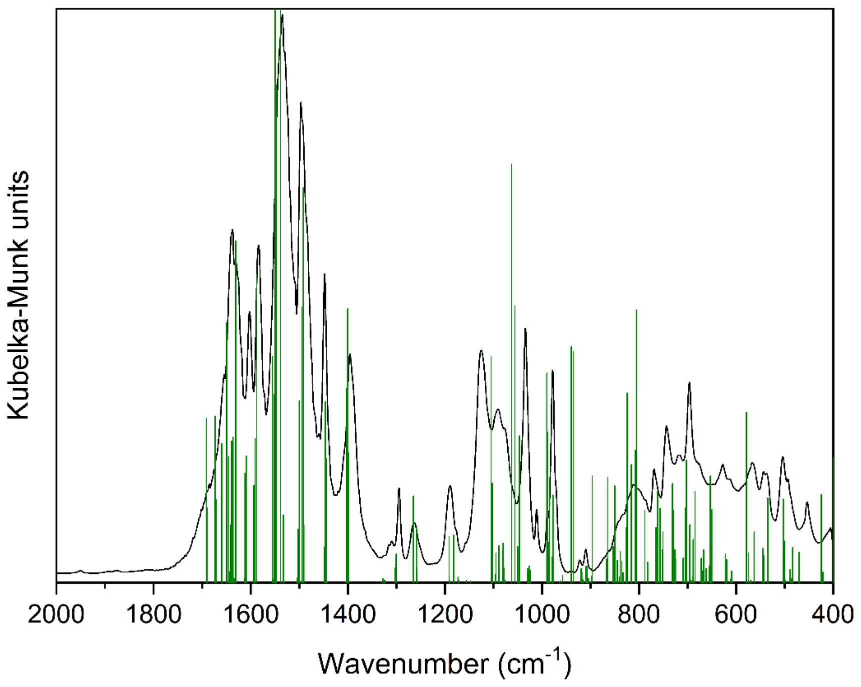

Figure S10. The comparison of calculated (CRYSTAL17 programme) vibrational modes using B3LYP (green lines) functional with recorded DRIFTS spectrum of **phbigua$_2$HPO$_3$**.

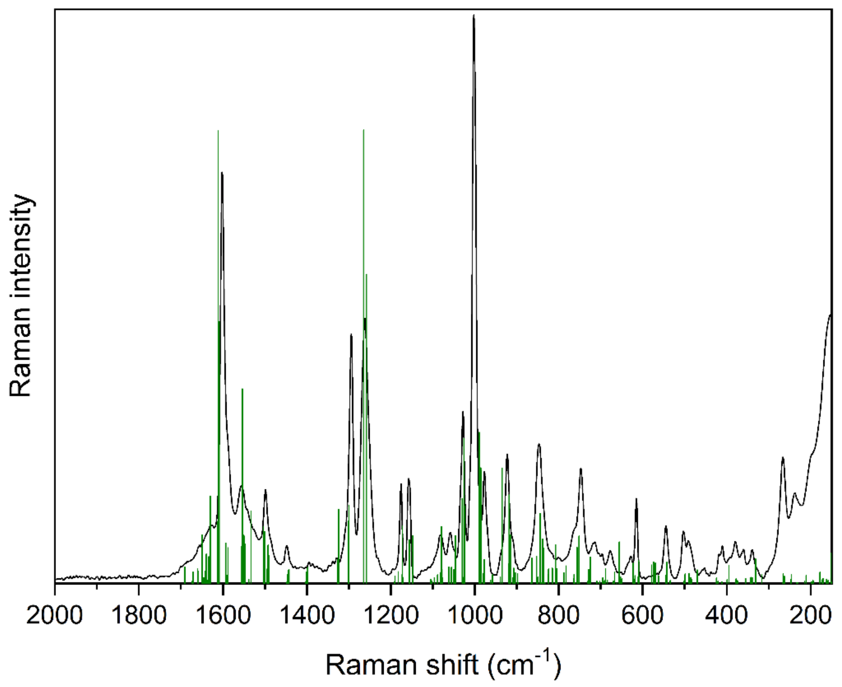

Figure S11. The comparison of calculated (CRYSTAL17 programme) vibrational modes using B3LYP (green lines) functional with recorded Raman spectrum of **phbigua$_2$HPO$_3$**.

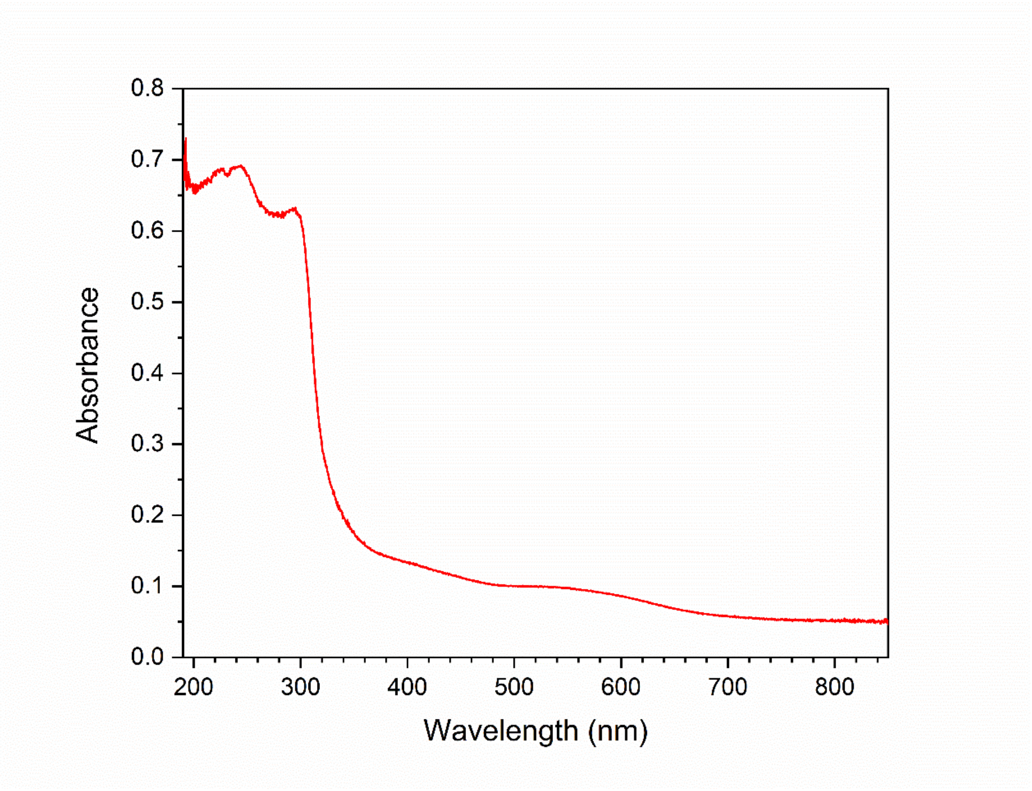

Figure S12. UV-Vis spectrum of powdered **phbigua$_2$SO$_4$**.

Table S10. Experimental powder diffraction data for **phbiguaNO₃**.

2 Theta (°)	d (Å)	Intensity (%)	2 Theta (°)	d (Å)	Intensity (%)
8.78	10.06	100	25.46	3.50	6
9.41	9.39	5	26.24	3.39	17
13.83	6.40	28	26.43	3.37	43
16.04	5.52	6	26.55	3.35	52
17.62	5.03	16	27.64	3.23	2
17.95	4.94	10	27.88	3.20	2
18.60	4.77	5	28.44	3.14	12
18.90	4.69	14	29.26	3.05	3
19.59	4.53	1	29.59	3.02	16
20.28	4.38	8	30.16	2.96	11
21.11	4.20	14	31.10	2.87	5
21.26	4.18	22	31.22	2.87	4
22.78	3.90	59	35.51	2.53	3
23.00	3.86	11	35.69	2.52	2
23.84	3.73	11	45.06	2.01	1

Table S11. Experimental powder diffraction data for **phbiguaClO₄**.

2 Theta (°)	d (Å)	Intensity (%)	2 Theta (°)	d (Å)	Intensity (%)
9.84	8.99	16	23.94	3.71	14
9.97	8.86	18	24.19	3.68	14
15.28	5.79	20	25.17	3.54	67
16.22	5.46	21	26.05	3.42	12
17.67	5.01	23	26.25	3.39	9
18.98	4.67	38	26.67	3.34	7
19.17	4.63	26	27.08	3.29	10
19.43	4.56	44	27.16	3.28	10
19.98	4.44	100	27.87	3.20	7
20.36	4.36	13	29.53	3.02	7
20.52	4.33	16	30.13	2.96	9
20.92	4.24	23	30.71	2.91	12
22.35	3.97	59	31.54	2.83	10
23.33	3.81	10	33.25	2.69	7
23.61	3.77	11	33.58	2.67	10

Table S12. Experimental powder diffraction data for **phbiguaHCO$_3$**.

2 Theta (°)	d (Å)	Intensity (%)	2 Theta (°)	d (Å)	Intensity (%)
8.78	10.07	100	23.62	3.77	2
10.74	8.24	1	25.32	3.52	1
13.46	6.58	6	26.51	3.36	58
14.54	6.09	22	29.47	3.03	12
15.10	5.87	14	30.07	2.97	4
15.85	5.59	2	30.68	2.91	5
16.28	5.45	20	31.13	2.87	2
17.60	5.04	23	31.48	2.84	5
18.35	4.83	14	31.85	2.81	2
20.09	4.42	2	33.51	2.67	2
20.62	4.31	2	34.23	2.62	5
21.40	4.15	10	35.17	2.55	2
21.88	4.06	52	35.58	2.52	4
22.51	3.95	6	36.58	2.46	2
23.19	3.84	3	39.94	2.26	2

Table S13. Experimental powder diffraction data for **phbigua$_2$SO$_4$**.

2 Theta (°)	d (Å)	Intensity (%)	2 Theta (°)	d (Å)	Intensity (%)
7.77	11.37	1	26.62	3.35	9
9.45	9.35	100	27.45	3.25	2
11.48	7.71	2	28.52	3.13	12
14.79	5.99	11	28.75	3.10	13
15.99	5.54	12	29.24	3.05	7
16.72	5.30	6	29.58	3.02	11
16.78	5.28	2	30.53	2.93	1
18.04	4.91	50	32.38	2.77	3
18.99	4.67	1	34.16	2.62	1
20.27	4.38	12	34.71	2.58	1
20.40	4.35	16	38.49	2.34	2
20.98	4.23	2	39.90	2.26	1
21.75	4.08	80	42.64	2.12	2
23.01	3.86	5	43.89	2.06	1
26.16	3.41	3	45.28	2.00	1

Table S14. Experimental powder diffraction data for **phbigua$_2$HPO$_4$1.5H$_2$O**.

2 Theta (°)	d (Å)	Intensity (%)	2 Theta (°)	d (Å)	Intensity (%)
6.72	13.14	32	19.61	4.52	23
7.38	11.97	29	21.96	4.04	47
9.58	9.22	81	22.16	4.01	27
9.66	9.15	100	22.82	3.89	22
12.96	6.83	20	23.42	3.80	53
13.26	6.67	15	24.43	3.64	16
14.08	6.28	18	24.87	3.58	16
16.37	5.41	15	25.32	3.51	33
16.88	5.25	12	27.59	3.23	22
17.11	5.18	27	28.48	3.13	31
17.40	5.09	21	28.83	3.09	22
18.07	4.90	16	29.13	3.06	18
18.84	4.71	19	29.34	3.04	15
19.13	4.64	52	31.84	2.81	17
19.32	4.59	76	32.03	2.79	25

Table S15. Experimental powder diffraction data for **phbigua$_2$HPO$_3$**.

2 Theta (°)	d (Å)	Intensity (%)	2 Theta (°)	d (Å)	Intensity (%)
10.26	8.61	31	24.43	3.64	21
10.90	8.11	17	25.22	3.53	45
13.98	6.33	12	26.65	3.34	14
14.81	5.98	13	28.05	3.18	45
15.12	5.86	10	28.33	3.15	42
15.70	5.64	43	29.50	3.03	32
18.02	4.92	63	30.65	2.92	21
18.46	4.80	15	31.63	2.83	17
20.15	4.40	100	32.10	2.79	19
21.32	4.16	64	32.58	2.75	23
21.55	4.12	63	34.13	2.62	13
22.50	3.95	46	34.75	2.58	11
22.02	4.03	19	35.43	2.53	10
23.10	3.85	16	38.09	2.36	12
23.38	3.80	10	39.57	2.28	10

Table S16. Experimental powder diffraction data for **phbigua$_2$HPO$_3$2H$_2$O**.

2 Theta (°)	d (Å)	Intensity (%)	2 Theta (°)	d (Å)	Intensity (%)
7.28	12.14	12	20.39	4.35	48
7.57	11.67	17	21.06	4.22	69
9.43	9.37	28	21.46	4.14	36
10.06	8.79	41	22.00	4.04	13
10.68	8.28	25	22.31	3.98	50
13.80	6.41	20	24.28	3.66	19
14.33	6.18	12	24.97	3.56	47
14.57	6.07	17	27.81	3.21	49
15,14	5.85	19	27.91	3.19	36
15.44	5.73	59	28.11	3.17	49
17.24	5.14	14	28.50	3.13	22
17.82	4.97	82	29.32	3.04	25
19.75	4.49	43	30.47	2.93	16
19.92	4.45	100	31.84	2.81	16
20.19	4.40	46	32.41	2.76	20
